# PPAR agonists as add-on treatment with metformin in management of type 2 diabetes: a systematic review and meta-analysis

**DOI:** 10.1038/s41598-024-59390-z

**Published:** 2024-04-16

**Authors:** Saif Alnuaimi, Tea Reljic, Fatima S. Abdulla, Hamda Memon, Sarah Al-Ali, Teagen Smith, Fadila Serdarevic, Zelija Velija Asimi, Ambuj Kumar, Sabina Semiz

**Affiliations:** 1 College of Medicine and Health Sciences, Khalifa University, PO Box 127788, Abu Dhabi, United Arab Emirates; 2 Research Methodology and Biostatistics Core, Morsani College of Medicine, University of South Florida, Tampa, FL USA; 3 Sarajevo Medical School, University Sarajevo School of Science and Technology, Sarajevo, Bosnia and Herzegovina; 4 Department of Child and Adolescent Psychiatry, Erasmus University Medical Center, Rotterdam, The Netherlands

**Keywords:** Glycemic control, Insulin resistance, Lipids, Safety profile, Gastrointestinal intolerance, Diseases, Endocrinology, Health care, Medical research

## Abstract

The combination of metformin and the peroxisome proliferator-activated receptors (PPAR) agonists offers a promising avenue for managing type 2 diabetes (T2D) through their potential complementary mechanisms of action. The results from randomized controlled trials (RCT) assessing the efficacy of PPAR agonists plus metformin versus metformin alone in T2D are inconsistent, which prompted the conduct of the systematic review and meta-analysis. We searched MEDLINE and EMBASE from inception (1966) to March 2023 to identify all RCTs comparing any PPAR agonists plus metformin versus metformin alone in T2D. Categorical variables were summarized as relative risk along with 95% confidence interval (CI). Twenty RCTs enrolling a total of 6058 patients met the inclusion criteria. The certainty of evidence ranged from moderate to very low. Pooled results show that using PPAR agonist plus metformin, as compared to metformin alone, results in lower concentrations of fasting glucose [MD = − 22.07 mg/dl (95% CI − 27.17, − 16.97), HbA1c [MD = − 0.53% (95% CI − 0.67, − 0.38)], HOMA-IR [MD = − 1.26 (95% CI − 2.16, − 0.37)], and fasting insulin [MD = − 19.83 pmol/L (95% CI − 29.54, − 10.13)] without significant increase in any adverse events. Thus, synthesized evidence from RCTs demonstrates the beneficial effects of PPAR agonist add-on treatment versus metformin alone in T2D patients. In particular, novel dual PPARα/γ agonist (tesaglitazar) demonstrate efficacy in improving glycaemic and lipid concentrations, so further RCTs should be performed to elucidate the long-term outcomes and safety profile of these novel combined and personalized therapeutic strategies in the management of T2D.

PROSPERO registration no. CRD42023412603.

## Introduction

Type 2 diabetes mellitus is a chronic disease characterized by insulin resistance and inadequate pancreatic insulin secretion, resulting in hyperglycemia, and requiring continuous medical care. Approximately 462 million people (6.3% of the global population) were affected by type 2 diabetes in 2017^1^ and it is expected that about 643 million people (11.3% of the global population) will be diagnosed with diabetes by 2030^2^. The management of type 2 diabetes involves a multifaceted approach, including lifestyle modifications, pharmacotherapy, and personalized treatment approach. Current clinical practice recommendations by the American Diabetes Association (ADA) recommends metformin as the first-line therapy, with sulfonylurea, thiazolidinedione (TZD), alpha-glucosidase inhibitors, benzoic acid derivatives, glucagon-like peptide 1 receptor agonists, dipeptidyl peptidase-4 inhibitors, and sodium glucose cotransporter 2 inhibitors as the second-line treatment options, often included in combination therapy^3,4^. By using drugs with different mechanisms of action, the diverse mechanisms responsible for progression of type 2 diabetes can be addressed, including the management of hyperlipidemia, hypertension, and other related micro- and macrovascular complications. In line with these recent ADA’s Standards of Care recommendations, the selection of drugs added to metformin should be based on the clinical characteristics, including the presence/risk of atherosclerotic cardiovascular disease, heart failure, chronic kidney disease, obesity, nonalcoholic fatty liver disease or nonalcoholic steatohepatitis, and risk for specific adverse drug effects^4^.

The combination of metformin with peroxisome proliferator-activated receptor (PPAR) agonists has garnered noteworthy attention due to the potential beneficial effects on metabolic control and safety profile through their potential complementary mechanisms of action. Metformin’s glucose-lowering effects are related to reduced mitochondrial respiration, lower hepatic energy production, and decreased glucose production by hepatic cells^5,6^. PPAR agonists, on the other hand, activate PPARα and/or PPARγ receptors, influencing insulin sensitivity and lipid metabolism. The activation of PPARα decreases triglyceride concentrations, while the activation of PPARγ leads to insulin sensitization and enhanced glucose metabolism^7^. The fibrate class of hypolipidemic drugs activates PPARα, while antidiabetic agents thiazolidinediones (glitazones) activate PPARγ receptor-regulated pathways, such as adipogenesis, lipid metabolism, glucose control and inflammation^8^, as well as demonstrate other pleiotropic effects^9^. Although TZDs, such as troglitazone and rosiglitazone, lost their approvals due to severe side effects, including CV risk, hepatotoxicity, bone fractures, and bladder cancer, pioglitazone showed cardiovascular benefits^10^. A recent systematic review and meta-analysis demonstrated no significant effects of pioglitazone on incident major adverse cardiovascular events, all-cause mortality, and hospitalization for heart failure^11^. Furthermore, it was shown that liver steatosis, inflammation, and insulin resistance were improved in patients with type 2 diabetes following one-year pioglitazone treatment^12^.

Similarly, dual-acting PPARα/γ agonists (glitazars), such as tesaglitazar, also beneficially affected the glucose metabolism, insulin resistance and atherogenic dyslipidaemia in patients with type 2 diabetes^13–15^. In addition to their beneficial effects in lowering glucose and triglyceride concentrations^16^, PPARα/γ agonists have been also associated with adverse effects, including myocardial ischemia and congestive heart failure^17,18^, which led to the discontinued use for most of these drugs^18^. Saroglitazar is the first approved novel dual PPARα/γ agonist that demonstrated efficacy in improving glycaemic and lipid concentrations in patients with diabetic dyslipidemia, with the relative absence of adverse events^19–21^.

Thus, in line with the person-centered diabetes care recommendations^4^, the combination of metformin and PPAR agonists might be of interest in the treatment of diabetic patients who, based on their clinical characteristics, would benefit from the reported effects of these oral antidiabetic drugs. Previous studies indicated that insulin resistance was more attenuated upon combined treatment of rosiglitazone and metformin as compared to metformin-treated type 2 diabetic patients^22,23^. Furthermore, the combination of pioglitazone with metformin was reported to lead to better control of HbA1c and lipid concentrations as compared to diabetic patients who were treated with metformin only^24,25^. Interestingly, the results of the previous double-blind randomized controlled trial (RCT) showed that add-on treatment with dual PPARα/γ agonist, muraglitazar, resulted in greater improvement in HbA1c and lipid concentrations than when pioglitazone was added to metformin^26^. However, the weight gain and edema were more common in patients who were treated with combined treatment of metformin and muraglitazar as compared with an addition of pioglitazone to metformin therapy^26^.

Accordingly, here we performed a systematic review and meta-analysis where we aimed to assess the efficacy of combined treatment of metformin plus PPAR agonists versus metformin treatment alone in improving glycemic control, lipid profile, and adverse events in patients with type 2 diabetes. Our main objective is to synthesize all available evidence to assess the benefits and risks associated with the combined treatment of metformin and PPAR agonists versus metformin alone in the management of type 2 diabetes. The specific question was the following: In adults (≥ 18 years of age) with type 2 diabetes, does combination of any PPAR agonists plus metformin compared with metformin alone result in improved primary (fasting glucose [FG] and HbA1c) and secondary outcomes (fasting insulin [FI], Homeostatic Model Assessment for Insulin Resistance [HOMA-IR], Homeostatic Model Assessment for Beta-cell function [HOMA-B], High-sensitivity C-reactive protein [hs-CRP], high-density lipoprotein cholesterol [HDL-C], low-density lipoprotein cholesterol [LDL-C], total cholesterol [TC], triglycerides [TG], systolic blood pressure [SBP], and diastolic blood pressure [DBP]) without increased risk of adverse events (any and gastrointestinal) associated with the treatments in an outpatient setting?

## Methods

This systematic review was performed according to a pre-specified protocol and the standard methods in Cochrane Handbook for Systematic Reviews of Interventions and is reported according to Preferred Reporting Items for Systematic Reviews and Meta-Analyses (PRISMA) guidelines^27,28^. The protocol for this systematic review was registered in PROSPERO (CRD42023412603).

### Selection criteria

Any randomized control trial (RCT) enrolling adult patients with type 2 diabetes assessing the efficacy of any PPAR agonists plus metformin versus metformin alone was eligible for inclusion. RCTs in pediatric population or observational study designs were not eligible for inclusion. There were no restrictions on the inclusion according to the language of the publication, location, or date of study.

### Outcome measures

The primary outcomes were fasting glucose (FG, mg/dl) and hemoglobin A1C (%) concentrations. The secondary outcomes were fasting insulin (FI, pmol/L), HOMA-IR, HOMA-B, hsCRP, high-density lipoprotein cholesterol (HDL-C, mg/dl), low-density lipoprotein cholesterol (LDL-C, mg/dl), total cholesterol (TC, mg/dl), and triglycerides (TG, mg/dl) as well as systolic blood pressure (SBP), diastolic blood pressure (DBP) and occurrence of any and gastrointestinal (GI) adverse effects.

### Search methods

A comprehensive and systematic search of PubMed and EMBASE databases was performed from inception until March 29, 2023. The complete search strategy for the two databases is illustrated in “Supplementary Appendix”. There were no limits for language. Furthermore, references of relevant review articles and included studies were hand searched to identify additional eligible studies.

### Data collection and analysis

All the citations obtained from the search was imported into EndNote software^29^. The duplicate citations were removed using the deduplication function in EndNote program. All the unique citations post deduplication were uploaded into Rayyan citation manager^30^. Two blinded review authors independently reviewed all titles, abstracts, and full-text reports to determine the eligibility of each reference for the inclusion in the systematic review as per the inclusion criteria using the Rayyan citation manager. Any disagreement in the inclusion was reviewed by the senior authors and resolved by consensus.

### Data extraction and management

Two review authors independently extracted data using a paper based standardized data extraction form from all included studies. Data were collected on study characteristics (study design, setting), participant characteristics (number of participants enrolled, age,), intervention: characteristics (PPAR agonist, dose, route, administration schedule, and associated therapies) and outcomes. We did not consider any imputation methods for missing data. We had plans to contact the corresponding author in case of unextractable data. However, following standard approaches we were able to extract data from all reported outcomes. All abstracted data were entered into the Review Manager Package^31^.

### Assessment of risk of bias in included studies

The risk of bias in the included studies was assessed using the Cochrane Risk of Bias assessment tool for RCT^32^. This tool includes the assessment of the method of randomization, allocation concealment, performance bias, detection bias, attrition bias, reporting bias, and any other bias. The overall certainty of evidence was assessed and summarized using the Grading of Recommendations Assessment, Development and Evaluation (GRADE) method. This method separates the quality of evidence based on the risk of bias, inconsistency of results, indirectness of evidence, imprecision, and reporting bias^33^.

### Assessment of heterogeneity and reporting biases

Heterogeneity between pooled studies was assessed using the I^2^ statistic. An I^2^ value of 0–40% might not be important; 30–60% may represent moderate heterogeneity, 50–90%: may represent substantial heterogeneity; and 75–100% considerable heterogeneity^28^.

### Statistical analysis

All continuous data prior to analysis were converted into the same metric using the online Omni Health Calculator^34^. All analyses were performed following the intention-to-treat principle. In studies with multiple arms, we divided in half the subjects in the control groups when comparing against experimental arms. Continuous data were summarized as mean difference (MD) along with 95% confidence interval (CI) for each study. Dichotomous data were summarized as risk ratio (RR) along with a 95% CI for each study. When appropriate, summary estimates from individuals studies were pooled under a random-effects model using the DerSimonian-Laird approach outlined a priori in a protocol^35^. We decided a priori to use random effects model as the model of choice because it is more conservative compared with fixed effects and also incorporates the between-study variance into the calculation. We planned for stratified analysis by PPAR agonist type only. We did not plan for any other subgroup analysis, meta-regression or assessment of publication bias. All data analyses were performed using Review Manager package^31^.

## Results

### Results of the search

**Figure 1:**
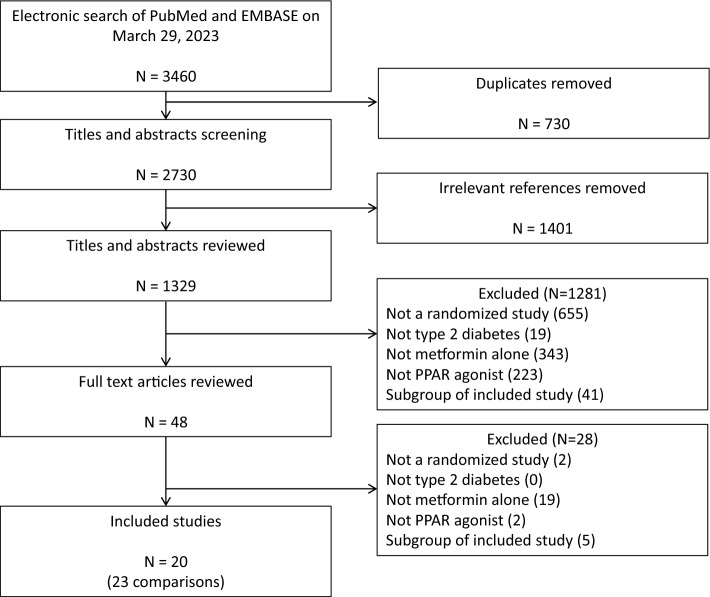
Study selection flow diagram.

The search strategy identified a total of 3460 citations. As shown in Fig. 1, after applying the inclusion criteria, 20 RCTs involving 23 comparisons and enrolling a total of 6529 patients met the inclusion criteria^22–25,36–51^.

### Characteristics of included studies

The total number of participants enrolled was 6058, with 3204 randomized to PPAR agonist plus metformin and 2854 randomized to the metformin-alone arm. As shown in Table 1, 85% of studies (17/20) reported a single comparison^22–25,36–40,42,45–51^, while the remaining 15% of studies (3/20) reported two comparison arms^41,43,44^. The daily metformin dose in the included studies ranged from 500 to 2550 mg/day. Pioglitazone was studied in 40% of studies (8/20) and the daily dose ranged from 15 to 45 mg/day^24,25,36,39,40,42,46,50^. Rosiglitazone was studied in 55% of studies (11/20) and the daily dose ranged from 4 to 8 mg/day^22,23,37,38,41,44,45,47–49,51^. Tesaglitazar was studied in 5% of studies (1/20) and the daily dose ranged from 0.5 to 1 mg/day^43^. The study duration ranged from 8 to 80 weeks. A majority of studies (60%, 12/20) evaluated HbA1c as their primary outcome^25,36–38,41,43,44,46–49,51^. A majority of studies (70%, 14/20) were sponsored by the pharmaceutical industry^24,25,36–38,40–43,46–49,51^, 10% (2/20) were sponsored by government/academia^22,45^, and 20% of studies (4/20) did not report the source of funding^23,39,44,50^.

**Table 1:** Study characteristics.

Study ID	Total number of patients	Metformin plus PPAR agonist arm	Metformin alone arm	Metformin dose	PPAR agonist	PPAR agonist dose	Mean age (MET/MET + PPAR)	Gender (Male/Female)	Location of trial	Single/Multicenter trial	Number of study arms	Study duration (weeks)	Primary outcome	A priori sample size calculations reported	Funding source
Bailey et al.^37^	569	289	280	2500 mg/day	Rosiglitazone	4 mg/day	57.6/58.1	327/241	Europe	Multicenter	2 arms	24	Hemoglobin A1c	Yes	GlaxoSmithKline
Borges et al.^38^	688	348	340	2000 mg/d	Rosiglitazone	4 mg/day	50.7/51.5	360/318	Global	Multicenter	2 arms	80	Hemoglobin A1c	Yes	GlaxoSmithKline
Derosa et al.^39^	136	69	67	3000 mg/d	Pioglitazone	45 mg/day	55/57	68/68	Italy	Multicenter	4 arms enrolled, 2 included in SR	64	BMI, HbA1c, FPG, PPG, FPI, PPI, GIR, and TGR	Yes	Not reported
Einhorn et al.^40^	328	168	160	stable dose for 30 days	Pioglitazone	30 mg/day	55.7/55.5	188/140	United States	Multicenter	2 arms	16	Not reported	Not reported	Takeda
Fonseca et al.^41^	348	119	116	2500 mg/day	Rosiglitazone	4 mg/day	58.8/57.5	231 /108	United States	Multicenter	3 arms	26	Hemoglobin A1c	Yes	GlaxoSmithKline
		113				8 mg/day	58.8/58.3								
Genovese et al.^42^	213	110	103	2550 mg/day	Pioglitazone	30 mg/day	57.8/57	127/86	Italy	Multicenter	2 arms	24	serum HDL cholesterol	Not reported	Takeda
Goke et al.^43^	590	194	200	2000 to 2500 mg/day	Tesaglitazar	0.5 mg/day	60.1/59.1	332/258	Global	Multicenter	3 arms	24	Hemoglobin A1c	Yes	AstraZeneca
		196				1 mg/day	60.1/58.4								
Gomez-Perez et al.^44^	116	37	39	2500 mg/day	Rosiglitazone	4 mg/day	53.4/51.7	27/78	Mexico	Multicenter	3 arms	26	Hemoglobin A1c	Yes	Not reported
		40				8 mg/day	53.4/54.2								
Hanefeld et al.^24^	81	39	42	1700 mg/day	Pioglitazone	30 mg/day	64.2/63.3	49/22	Germany	Multicenter	3 arms enrolled, 2 included in SR	24	Matrix Metallo Proteinase 9	Yes	Takeda
Kadoglou et al.^22^	100	50	50	850 to 2550 mg/day	Rosiglitazone	8 mg/day	62.7/62	29/68	Greece	Single center	2 arms	14	Emergence of novel cardiovascular risk factors	Not reported	European Social Fund and National Resources and Aristotle University Of Thessaloniki
Kadoglou et al.^45^	140	70	70	1700 mg/day	Rosiglitazone	4 mg/day	62.7/62	37/99	Greece	Not reported	2 arms	24	Serum adipokine	Not reported	European Social Fund and National Resources and Aristotle University Of Thessaloniki
Kaku et al.^25^	169	83	86	500 or 700 mg/day	Pioglitazone	15 mg/day	53/52	104/65	Japan	Multicenter	2 arms	28	Hemoglobin A1c	Yes	Takeda
Negro et al.^23^	38	19	19	up to 2550 mg/day	Rosiglitazone	8 mg/day	59/60.3	22/16	Italy	Not reported	2 arms	52	Not reported	Not reported	Not reported
Perez et al.^46^	411	201	210	1700 mg/day	Pioglitazone	30 mg/day	53.7/54.7	188/223	Multinational	Multicenter	3 arms enrolled, 2 included in SR	24	Hemoglobin A1c	Yes	Takeda
Rosenstock et al.^47^	309	155	154	2000 mg/day	Rosiglitazone	8 mg/day	51.5/50.1	176/133	Global	Multicenter	3 arms enrolled, 2 included in SR	32	Hemoglobin A1c	Yes	GlaxoSmithKline
Scott et al.^48^	179	87	92	≥ 1500 mg/day	Rosiglitazone	8 mg/day	55.3/54.8	109/70	Multinational	Multicenter	3 arms enrolled, 2 included in SR	18	Hemoglobin A1c	Not reported	Merck
Stewart et al.^49^	526	254	272	500 mg/day	Rosiglitazone	8 mg/day	59/58.9	290/236	Europe	Multicenter	2 arms	32	Hemoglobin A1c	Yes	GlaxoSmithKline
Takeda^36^	315	157	158	1000 mg/day	Pioglitazone	30 mg/day	57.6/56.7	133/179	Americas	Multicenter	2 arms	16	Hemoglobin A1c	Not reported	Takeda
Wang et al.^50^	36	24	12	1000 or 1500 mg/day	Pioglitazone	30 mg/day	58/60	17/19	Not reported	Not reported	2 arms	8	Not reported	Not reported	Not reported
Weissman et al.^51^	766	382	384	2000 mg/day	Rosiglitazone	8 mg/day	55.7/55.5	362/347	United States	Multicenter	2 arms	24	Hemoglobin A1c	Yes	GlaxoSmithKline

### Assessment of methodological quality of included studies

As shown in Supplement Fig. 1, of the 20 RCTs, for the generation of randomization sequence, 5 (25%) RCTs were rated as low risk of bias^22,37,39,41,50^, and 15 (75%) as unclear risk of bias^23–25,36,38,40,42–49,51^. For the adequacy of allocation concealment, 3 RCTs (15%) were rated as low risk of bias^22,39,41^, and 17 (85%) as unclear risk of bias^23–25,36–38,40,42–49,51^. For the blinding of participants and personnel, 17 (85%) RCTs were rated as low risk of bias^23–25,36–43,46–49,51,52^, 2 (10%) RCTs as high risk and 1 (5%) as unclear risk of bias^44,45^. For the blinding of outcomes assessors, 16 (80%) RCTs were rated as low risk of bias^23–25,36–43,46,47,49,51,52^, 1 (5%) as high risk^45^, and 3 (15%) as unclear risk of bias^22,44,48^. For the domain of incomplete outcome data, 1 RCT (5%) was rated as low risk of bias^42^, 17 (85%) RCTs as high risk^22,24,25,36–41,43–49,51^, and 2 (10%) RCTs as unclear risk^23,50^. For the domain of selective reporting of outcome, 18 (90%) RCTs were rated as low risk of bias^22–25,36–45,47–49,51^ and 2 (10%) RCTs as high risk^46,52^. All RCTs were rated as low risk of bias for other biases^22–25,36–49,51,53^. The overall certainty of evidence of included RCTs ranged from very low to moderate (Table 2).

**Table 2:** Summary of findings.

PPAR agonist plus metformin compared to metformin alone for type 2 Diabetes Mellitus					
Patient or population: Type 2 Diabetes Mellitus					
Intervention: PPAR agonist plus metformin					
Comparison: metformin alone					
Outcomes	No of participants (studies) Follow-up	Certainty of the evidence (GRADE)	Relative effect (95% CI)	Anticipated absolute effects	
				Risk with metformin alone	Risk difference with PPAR agonist plus metformin
Fasting glucose	5647 (22 RCTs)	⨁⨁◯◯ Low^a,b^	–	The mean fasting glucose was 169.9 mg/dl	MD 22.02 lower (2717 lower to 16.97 lower)
HbA1c	5611 (21 RCTs)	⨁⨁◯◯ Low^a,b^	–	The mean HbA1C was 7.95%	MD 0.53 lower (0.67 lower to 0.38 lower)
HOMA-IR	875 (7 RCTs)	⨁◯◯◯ Very low^a,b,c^	–	The mean HOMA-IR was 4.68	MD 1.26 lower (2.16 lower to 0.37 lower)
Fasting insulin	3434 (16 RCTs)	⨁◯◯◯ Very low^a,b,c^	–	The mean fasting insulin was 91.8 pmol/L	MD 19.83 lower (29.54 lower to 10.13 lower)
Total cholesterol	5607 (21 RCTs)	⨁⨁◯◯ Low^a,b^	–	The mean total cholesterol was 195.9 mg/dl	MD 10.7 higher (7.19 higher to 13.95 higher)
LDL-C	5569 (20 RCTs)	⨁⨁◯◯ Low^a,b^	–	The mean LDL-C was 116.5 mg/dl	MD 6.81 higher (3.28 higher to 10.33 higher)
Any adverse event	4841 (16 RCTs)	⨁⨁⨁◯ Moderate^a^	RR 1.02 (0.97 to 1.08)	55 per 100	1 more per 100 (2 fewer to 4 more)
Gastrointestinal adverse events	4083 (10 RCTs)	⨁⨁⨁◯ Moderate^a^	RR 0.81 (0.73 to 0.90)	29 per 100	5 fewer per 100 (8 fewer to 3 fewer)

*The risk in the intervention group (and its 95% confidence interval) is based on the assumed risk in the comparison group and the relative effect of the intervention (and its 95% CI).

CI: confidence interval; MD: mean difference; RR: risk ratio.

GRADE Working Group grades of evidence.

High certainty: we are very confident that the true effect lies close to that of the estimate of the effect.

Moderate certainty: we are moderately confident in the effect estimate: the true effect is likely to be close to the estimate of the effect, but there is a possibility that it is substantially different.

Low certainty: our confidence in the effect estimate is limited: the true effect may be substantially different from the estimate of the effect.

Very low certainty: we have very little confidence in the effect estimate: the true effect is likely to be substantially different from the estimate of effect.

Explanations.

^a^Of all the included studies, the reporting of the method of randomization sequence generation and allocation conceal is unclear for several studies.

^b^Heterogeneity between studies is high.

^c^Confidence intervals for the pooled estimate are wide.

### Outcomes

#### Fasting glucose

**Figure 2:**
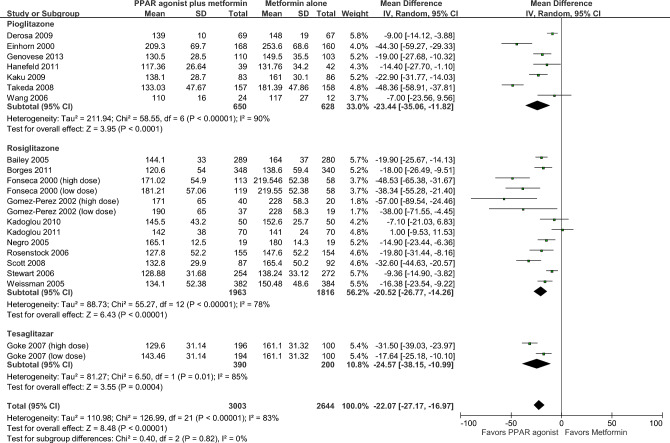
Fasting glucose.

As shown in Fig. 2, fasting glucose (FG) concentrations were reported in 19 RCTs (22 comparisons) enrolling 5647 patients^22–25,36–45,47–51^. The mean FG was significantly lower in patients treated with metformin plus PPAR agonist compared to patients treated with metformin alone (MD = − 22.07 mg/dl, 95% CI = − 27.17, − 16.97; *p* < 0.001). Heterogeneity among pooled RCTs was substantial (I^2^ = 83%). The overall certainty in the estimate was low (Table 2).

There was no significant difference between the subgroups (*p* = 0.82).

#### Hemoglobin A1c

**Figure 3:**
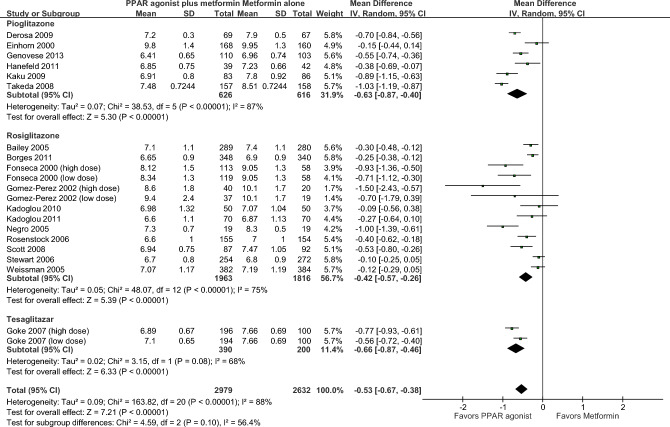
Hemoglobin A1c.

As shown in Fig. 3, hemoglobin A1c was reported in 18 RCTs (21 comparisons) enrolling 5611 patients^22–25,36–45,47–49,51^. The mean HbA1c concentrations were significantly lower in patients treated with metformin plus PPAR agonist compared to patients treated with metformin alone (MD = − 0.53%, 95% CI = − 0.67, − 0.38; *p* < 0.001). Heterogeneity among pooled RCTs was substantial (I^2^ = 88%). The overall certainty in the estimate was low (Table 2).

There was no significant difference between the subgroups (*p* = 0.10).

#### HOMA-IR

As shown in Supplement Fig. 2, HOMA-IR was reported in 7 RCTs (7 comparisons) enrolling 875 patients^22,23,25,42,45,48,50^. The mean HOMA-IR was significantly lower in patients treated with metformin plus PPAR agonist compared to patients treated with metformin alone (MD = − 1.26, 95% CI = − 2.16, − 0.37; *p* = 0.006). Heterogeneity among pooled RCTs was considerable (I^2^ = 91%). The overall certainty in the estimate was very low (Table 2).

There was no significant difference between the subgroups (*p* = 0.44).

#### Fasting insulin

As shown in Supplement Fig. 3, fasting insulin was reported in 14 RCTs (16 comparisons) enrolling 3434 patients^22–25,37,39,41–43,45,47–50^. The mean fasting insulin concentrations were significantly lower in patients treated with metformin plus PPAR agonist compared to patients treated with metformin alone (MD = − 19.83 pmol/L, 95% CI = − 29.54, − 10.13; *p* < 0.001). Heterogeneity among pooled RCTs was considerable (I^2^ = 93%). The overall certainty in the estimate was very low (Table 2).

There was no significant difference between the subgroups (*p* = 0.10).

#### HOMA-B

As shown in Supplement Fig. 4, HOMA-B was reported in 4 RCTs (4 comparisons) enrolling 1487 patients^37,42,48,49^. The mean HOMA-B was significantly higher in patients treated with metformin plus PPAR agonist compared to patients treated with metformin alone (MD = 7.45, 95% CI = 3.45, 11.45; *p* = 0.0003). Heterogeneity among pooled RCTs was substantial (I^2^ = 65%).

There was a significant difference between the subgroups (*p* = 0.009).

#### hsCRP

As shown in Supplement Fig. 5, hsCRP was reported in 9 RCTs (10 comparisons) enrolling 2520 patients^22,24,25,37,43,45,47,49,50^. The mean hsCRP concentrations were significantly lower in patients treated with metformin plus PPAR agonist compared to patients treated with metformin alone (MD = − 0.62 mg/L, 95% CI = − 0.87, − 0.37; *p* < 0.001). Heterogeneity among pooled RCTs was substantial (I^2^ = 76%).

There was no significant difference between the subgroups (*p* = 0.97).

#### Total cholesterol

As shown in Supplement Fig. 6, total cholesterol was reported in 18 RCTs (21 comparisons) enrolling 5607 patients^22–25,37,38,40–51^. The mean total cholesterol concentrations were significantly higher in patients treated with metformin plus PPAR agonist compared to patients treated with metformin alone (MD = 10.57 mg/dl, 95% CI = 7.19, 13.95; *p* < 0.001). Heterogeneity among pooled RCTs was considerable (I^2^ = 97%). The overall certainty in the estimate was low (Table 2).

There was a significant difference between the subgroups (*p* = 0.008).

#### High-density lipoprotein cholesterol

As shown in Supplement Fig. 7, HDL-cholesterol was reported in 17 RCTs (21 comparisons) enrolling 5607 patients^22,24,25,37,38,40–51^. The mean HDL-cholesterol concentrations were significantly higher in patients treated with metformin plus PPAR agonist compared to patients treated with metformin alone (MD = 2.81 mg/dl, 95% CI = 2.00, 3.62; *p* < 0.001). Heterogeneity among pooled RCTs was considerable (I^2^ = 94%).

There was no significant difference between the subgroups (*p* = 0.32).

#### Low-density lipoprotein cholesterol

As shown in Supplement Fig. 8, LDL-cholesterol was reported in 17 RCTs (20 comparisons) enrolling 5569 patients^22,24,25,37,38,40–51^. The mean LDL-cholesterol concentrations were significantly higher in patients treated with metformin plus PPAR agonist compared to patients treated with metformin alone (MD = 6.81 mg/dl, 95% CI = 3.28, 10.33; *p* = 0.0002). Heterogeneity among pooled RCTs was considerable (I^2^ = 98%). The overall certainty in the estimate was low (Table 2).

There was a significant difference between the subgroups (*p* = 0.0001).

#### Triglycerides

As shown in Supplement Fig. 9, triglycerides were reported in 18 RCTs (21 comparisons) enrolling 5507 patients^22–25,37,38,40–51^. There was no significant difference in mean triglycerides concentrations in patients treated with metformin plus PPAR agonist compared to patients treated with metformin alone (MD = − 10.96 mg/dl, 95% CI = − 22.10, 0.18; *p* = 0.05). Heterogeneity among pooled RCTs was considerable (I^2^ = 98%).

There was a significant difference between the subgroups (*p* = 0.003).

#### Systolic blood pressure

As shown in Supplement Fig. 10, systolic blood pressure (BP) was reported in 6 RCTs (6 comparisons) enrolling 1053 patients^22,23,42,45,49,50^. The mean systolic BP was significantly lower in patients treated with metformin plus PPAR agonist compared to patients treated with metformin alone (MD = − 3.19 mmHg, 95% CI = − 4.83, − 1.55; *p* = 0.0001). Heterogeneity among pooled RCTs was not important (I^2^ = 27%).

There was no significant difference between the subgroups (*p* = 0.64).

#### Diastolic blood pressure

As shown in Supplement Fig. 11, diastolic blood pressure (BP) was reported in 6 RCTs (6 comparisons) enrolling 1053 patients^22,23,42,45,49,50^. The mean diastolic BP was significantly lower in patients treated with metformin plus PPAR agonist compared to patients treated with metformin alone (MD = − 2.82 mmHg, 95% CI = − 4.99, − 0.64; *p* = 0.01). Heterogeneity among pooled RCTs was substantial (I^2^ = 81%).

There was no significant difference between the subgroups (*p* = 0.41).

#### Any adverse events

**Figure 4:**
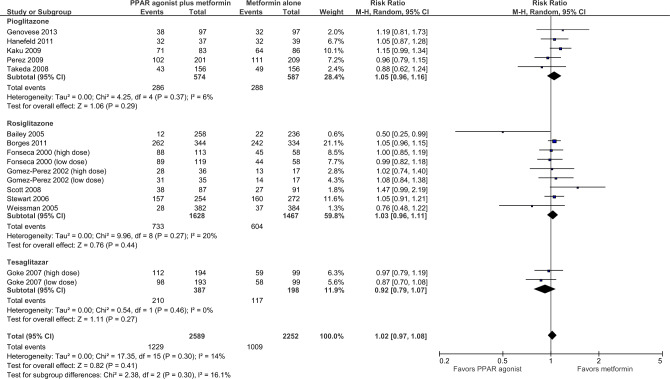
Any adverse event.

As shown in Fig. 4, adverse events were reported in 13 RCTs (16 comparisons) enrolling 4841 patients^24,25,36–38,41–44,46,48,49,51^. The risk of adverse events in patients treated with metformin plus PPAR agonist compared to patients treated with metformin alone was not significant (RR = 1.02, 95% CI = 0.97, 1.08; *p* = 0.41). Heterogeneity among pooled RCTs was not important (I^2^ = 14%). The overall certainty in the estimate was moderate (Table 2).

There was no significant difference between the subgroups (*p* = 0.30).

#### Gastrointestinal intolerance

As shown in Supplement Fig. 12, adverse events were reported in 9 RCTs (10 comparisons) enrolling 4083 patients^37,38,42,43,46–49,51^. Patients treated with metformin plus PPAR agonist had a significantly lower risk of gastrointestinal adverse events compared to patients treated with metformin alone (RR = 0.81, 95% CI = 0.73, 0.90; *p* < 0.001). The heterogeneity among pooled RCTs was not important (I^2^ = 0%). The overall certainty in the estimate was moderate (Table 2).

There was no significant difference between the subgroups (*p* = 0.31).

All results from the subgroup analyses by the agent are reported in the supplementary material.

## Discussion

The findings from our systematic review and meta-analysis, based on our knowledge, represent the largest body of synthesized evidence to date assessing the outcomes of metformin treatment alone versus combined treatment of metformin with PPAR agonists. The pooled results show that, on average, combination treatment with PPAR agonists compared with metformin alone is associated with significantly improved glycemic control in patients with type 2 diabetes. Specifically, the use of PPAR agonists plus metformin results in significantly lower concentrations of fasting glucose, hemoglobin A1c, fasting insulin, and HOMA-IR as compared to metformin treatment alone. In addition, the effect of combination treatment was consistent across all PPAR agonists types including PPARγ activators, pioglitazone and rosiglitazone, and dual PPARα/γ activator, tesaglitazar. These findings are in line with the previous studies that demonstrate the epidemiological and biological plausibility of these results. The beneficial effects of PPARα and PPARγ activation on glycemic control happens primarily by increasing insulin sensitivity and preserving beta-cell function^16,54^ and the activation of PPARγ improves insulin sensitization and glucose uptake^7,18^. Treatment with PPARγ agonists, TZDs, effectively lowers HbA1c concentrations by about 1% as monotherapy and improves insulin sensitivity in patients with type 2 diabetes^55^. Furthermore, the pioglitazone treatment lowered concentrations of fasting glucose, insulin, and HbA1c in type 2 diabetic patients^56^, while another TZD, rosiglitazone, improved overall glucose tolerance and increased insulin sensitivity in patients with impaired glucose tolerance and type 2 diabetes^57^. Previous studies have also reported decreased HOMA-IR index, glucose, insulin, and HbA1c concentrations in diabetic patients upon an addition of rosiglitazone^23^ and pioglitazone^25^ to metformin treatment. Also, rosiglitazone provided more durable glycemic control than metformin or sulfonylurea^58^. Similarly, another study showed that the addition of pioglitazone to metformin-treated type 2 diabetic patients decreased HbA1c and HOMA-IR^42^ as well as fasting insulin concentrations as compared with the sulfonylurea plus metformin group^59^.

The results of our meta-analysis demonstrated that treatment with PPARα/γ agonist, tesaglitazar, plus metformin reduced triglyceride (TG) concentrations in patients with type 2 diabetes, as compared to metformin treatment alone. However, treatment with TZDs (PPARγ agonists) plus metformin did not significantly affect TG concentrations. This is in line with the findings that the combined treatment of PPARα/γ agonist muraglitazar with metformin led to more enhanced effect in reducing TG concentrations as compared to the combined treatment of metformin with TZD agent pioglitazone^26^. Furthermore, results from recent clinical trials demonstrated that saroglitazar therapy decreased triglyceride concentrations by 45% as well as reduced concentrations of other atherogenic lipids, including TC, LDL-C, and VLDL-C^19,60^. It was also found that saroglitazar treatment improved lipid profile, including reduced TG, LDL-C, VLDL-C, TC, and increased HDL-C concentrations, in patients with type 2 diabetes receiving background metformin therapy^61^. The combined treatment of saroglitazar and metformin also resulted in a greater reduction of TG concentrations as compared to patients with type 2 diabetes who were treated with fenofibrate plus metformin^21^.

In addition to reduced triglyceride concentrations, our findings indicate that adding PPARα/γ activator (tesaglitazar) to metformin treatment does not significantly affect the concentrations of TC and LDL-C as compared to the patients with type 2 diabetes who were treated with metformin only. However, our meta-analysis demonstrates increased concentrations of total cholesterol and LDL-C upon treatment with PPARγ activators (TZDs) plus metformin vs metformin treatment alone. The results of the subgroup analysis per agent, showed that rosiglitazone plus metformin increased concentrations of TC and LDL-C, while pioglitazone plus metformin significantly affected TC concentrations only. This is in contrast to the previous studies which demonstrated that rosiglitazone has no significant effect on TG concentrations, while pioglitazone reduced TG and LDL particle size/concentrations^62^. Furthermore, it was reported that treatment with rosiglitazone plus metformin reduced concentrations of TG and TC^23^, while an addition of pioglitazone to metformin-treated patients with type 2 diabetes decreased TG, but increased HDL-C concentrations^59^. Since it was reported that metformin treatment itself reduces LDL-C concentrations in patients with type 2 diabetes^62,63^, it is possible that upon adding TZDs to metformin, the concentrations of LDL-C and/or TC concentrations increase as observed in our meta-analysis, which might be in line with the adverse effects of TZDs on the cardiovascular system. In line with our results, it was suggested that the potential difference in the risk of myocardial infarction between pioglitazone and rosiglitazone may lie in their different effects on lipoproteins concentrations, with pioglitazone demonstrating more favorable effects (TG decrease, HDL-C increase, with no effect on LDL-C or TC) than rosiglitazone (no effect on TG concentrations, HDL-C increase, but increases in LDL-C and TC concentrations)^64,65^. The pooled results as well as the results from the subgroup analysis according to the type of PPAR agonist showed the beneficial effects of the combined treatment of PPARα/γ or PPARγ agonists plus metformin vs metformin alone on HDL-C concentrations. This is in line with the previous studies, which also showed that increased HDL-C concentrations upon activation of PPARα^7,18^ and PPARγ receptors^59,66^.

Furthermore, our findings also indicated the beneficial effects of combined treatment of PPARγ agonists with metformin, which decreased systolic and diastolic blood pressure in patients with type 2 diabetes. This is in line with previous reports indicating that the activation of PPARγ lowers systemic blood pressure^7,18,22,45^. Another study also showed a reduction of systolic and diastolic blood pressure at 12 months of combined treatment with rosiglitazone and metformin, which correlated with HOMA-IR index, indicating that rosiglitazone can decrease blood pressure and that the enhancement of insulin sensitivity is associated with the reduction of blood pressure^23^.

Our findings showed that the concentrations of high-sensitivity C-reactive protein (hsCRP) were decreased following the combined treatment of PPARγ activators (TZDs) with metformin as compared to metformin treatment alone. This is in line with the previous studies, showing decreased concentrations of inflammation and cardiovascular risk markers, such as CRP, in obese and type 2 diabetic patients with TZD intervention^67^. Additionally, pioglitazone treatment significantly reduced CRP^56,68^ and hsCRP concentrations^52,59^. The addition of rosiglitazone to metformin resulted in reduced concentrations of hsCRP as compared to type 2 diabetes patients who were treated only with metformin^22,45^. It was also reported that the combined treatment of PPARα/γ agonist muraglitazar with metformin led to a more enhanced effect in reducing hsCRP concentrations as compared to combined treatment of metformin with pioglitazone. However, our results demonstrated that there was no significant effect on hsCRP concentrations when another agent from this class of dual PPARα/γ agonist, tesaglitazar, was added to metformin treatment.

Our results of overall and subgroup analysis showed that there were no significant side effects associated with the addition of any PPAR agonist to metformin in patients with type 2 diabetes as compared to metformin, which is contrast to a few studies reporting an increased risk of adverse events associated with the use of PPAR agonists^19,58,65,69,70^. However, most studies reported either any adverse event or gastrointestinal toxicities, so we could not compare the cardiac and other toxicities possibly associated with addition of PPAR agonists.

Metformin treatment is associated with a high incidence of gastrointestinal (GI) side effects^62^. Strikingly, our meta-analysis showed that the risk of GI events was reduced after adding PPARγ activator (TZDs) to metformin treatment, while this beneficial effect was not observed upon combined treatment with dual PPARα/γ activator vs metformin treatment alone.

There are a several limitations to this systematic review and meta-analysis. These limitations primarily relate to the conduct and reporting of individual RCTs included here, which may possibly affect the overall results. For example, the overall methodological quality of evidence ranged from very low to moderate due to risk of bias and heterogeneity in pooled estimates. The risk of bias assessment may possibly be a function of reporting and not necessarily conduct. Similarly, the reasons for heterogeneity could be multifactorial, including difference in primary outcomes, study duration, type of PPAR agonist, metformin and PPAR dosing across pooled studies. Most included RCTs did report the sample size assessment details, which possibly reduces the chance of random error, and we suspect that, given the consistency of effects observed across all glycemic outcomes, the results are possibly not influenced by random error and risk of bias. Another important issue limitation relates to generalizability. All RCTs in this systematic review assessed the efficacy of either PPARγ or α/γ agonists and therefore these findings are possibly limited to these specific types only and may not necessarily apply to PPARα or other PPAR agonists. However, our search did not find any RCTs assessing the efficacy of PPARα or other PPAR agonists. Once such RCTs are available this systematic review and meta-analysis will require an update. Nevertheless, despite these limitations, the impact of combination treatment with PPAR agonist compared with metformin alone in patients with type 2 diabetes is plausibly strong.

In conclusion, to the best of our knowledge, this meta-analysis is the largest body of synthesized evidence to date that assesses the outcomes of metformin treatment alone versus combined metformin and PPAR agonists in patients diagnosed with type 2 diabetes. Our findings indicate the beneficial effects of add-on treatment with PPAR agonist on glycemic control, reduced HbA1c concentrations, and ameliorated insulin resistance as compared to monotherapy with metformin. In addition, this combination also showed the favorable effects on type 2 diabetes-associated traits, including hypertension, increased concentrations of inflammatory markers, and dyslipidemia. Our meta-analysis demonstrated more favorable effects of combined metformin treatment with PPARα/γ activator (tesaglitazar) on lipid profile by lowering the concentrations of TG, increasing HDL-C concentrations and not increasing the concentrations of LDL-C and TC observed upon adding TZDs to metformin treatment. This might be in line with the adverse effects of TZDs on the cardiovascular system. However, our results also indicate that adding TZDs to metformin treatment results in a more favorable safety profile by reducing the number of GI adverse events as compared to type 2 diabetic patients who were treated with metformin only. Thus, it is crucial to consider the beneficial as well as the potential adverse events, such as gastrointestinal and cardiovascular events, which are related to use of the combination of metformin and PPAR agonists that would require monitoring and the potential adjustment of the prescribed medication or its dose. Further studies are warranted to elucidate the long-term outcomes and optimal usage of combined metformin and PPAR agonist in the management of type 2 diabetes, with continued research exploring optimal dosing regimens, long-term effects, and personalized treatment approaches. In addition, the potential for novel PPAR agonists with improved safety profiles warrants further investigation.

### Supplementary Information

Supplementary Information.

## Data Availability

All data generated or analyzed during this study are included in this published article and its supplementary files.

## Abbreviations

DBP: Diastolic blood pressure
FG: Fasting glucose
FI: Fasting insulin
GI: Gastrointestinal
HDL-C: High density lipoprotein-cholesterol
hsCRP: High-sensitivity C-reactive protein
LDL-C: Low density lipoprotein-cholesterol
MD: Mean difference
PPAR: Peroxisome proliferator-activated receptor
RCT: Randomized control trial
SBP: Systolic blood pressure
TC: Total cholesterol
TG: Triglycerides
TZD: Thiazolidinedione
