# Supplementary material for: PPAR agonists as add-on treatment with metformin in management of type 2 diabetes: a systematic review and meta-analysis
Source: Sci Rep. 2024 Apr 16;14:8809. doi: 10.1038/s41598-024-59390-z (PMC11021491; doi:10.1038/s41598-024-59390-z)

Supplementary Material: Results of the subgroup analysis by agent

Fasting glucose

Pioglitazone

Seven RCTs enrolling 1278 patients reported FG [24, 25, 33, 36, 37, 39, 47]. The mean FG was significantly lower in patients treated with pioglitazone plus metformin versus metformin alone (MD = -23.44 mg/dl, 95% CI = -35.06, -11.82; $p < 0.001$). Heterogeneity among pooled RCTs was substantial ($I^2 = 90\%$).

Rosiglitazone

Eleven RCTs (13 comparisons) enrolling 3779 patients reported FG [22, 23, 34, 35, 38, 41, 42, 44-46, 48]. The mean FG was significantly lower in patients treated with rosiglitazone plus metformin versus metformin alone (MD = -20.52 mg/dl, 95% CI = -26.77, -14.26; $p < 0.001$). Heterogeneity among pooled RCTs was substantial ($I^2 = 78\%$).

Tesaglitazar

One RCT (2 comparisons) enrolling 590 patients reported this outcome [40]. The mean FG was significantly lower in patients treated with tesaglitizar plus metformin versus metformin alone (MD = -24.57 mg/dl, 95% CI = -38.15, -10.99; $p = 0.0004$). Heterogeneity among pooled RCTs was substantial ($I^2 = 85\%$).

HbA1c

Pioglitazone

Six RCTs enrolling 1242 patients reported hemoglobin A1c [24, 25, 33, 36, 37, 39]. The mean HbA1c was significantly lower in patients treated with pioglitazone plus metformin versus

metformin alone (MD = -0.63%, 95% CI = -0.87, -0.40; $p < 0.001$). Heterogeneity among pooled RCTs was substantial ($I^2 = 87\%$).

Rosiglitazone

Eleven RCTs (13 comparisons) enrolling 3779 patients reported hemoglobin A1c [22, 23, 34, 35, 38, 41, 42, 44-46, 48]. The mean HbA1c was significantly lower in patients treated with rosiglitazone plus metformin versus metformin alone (MD = -0.42%, 95% CI = -0.57, -0.26; $p < 0.001$). Heterogeneity among pooled RCTs was substantial ($I^2 = 75\%$).

Tesaglitazar

One RCT (2 comparisons) enrolling 590 patients reported this outcome [40]. The mean hemoglobin A1c was significantly lower in patients treated with tesaglitizar plus metformin versus metformin alone (MD = -0.66%, 95% CI = -0.87, -0.46; $p < 0.001$). Heterogeneity among pooled RCTs was substantial ($I^2 = 68\%$).

HOMA-IR

Pioglitazone

Two RCTs enrolling 249 patients reported HOMA-IR [39, 47]. The mean HOMA-IR was significantly lower in patients treated with pioglitazone plus metformin versus metformin alone (MD = -1.69, 95% CI = -2.41, -0.97; $p < 0.00001$). The heterogeneity among pooled RCTs was not important ($I^2 = 0\%$).

Rosiglitazone

Five RCTs (5 comparisons) enrolling 626 patients reported HOMA-IR [22, 23, 25, 42, 45]. The mean HOMA-IR was significantly lower in patients treated with rosiglitazone plus metformin

versus metformin alone (MD = -1.17, 95% CI = -2.27, -0.08; p =0.04). Heterogeneity among pooled RCTs was considerable (I^2 = 93%).

Fasting insulin

Pioglitazone

Five RCTs enrolling 635 patients reported fasting insulin [24, 25, 36, 39, 47]. The mean fasting insulin was significantly lower in patients treated with pioglitazone plus metformin versus metformin alone (MD = -26.17 pmol/L, 95% CI = -40.45, -11.89; p =0.0003). Heterogeneity among pooled RCTs was substantial (I^2 = 75%).

Rosiglitazone

Eight RCTs (9 comparisons) enrolling 2209 patients reported fasting insulin [22, 23, 34, 38, 42, 44-46]. The mean fasting insulin was significantly lower in patients treated with rosiglitazone plus metformin versus metformin alone (MD = -13.59 pmol/L, 95% CI = -28.06, 0.89; p =0.07). Heterogeneity among pooled RCTs was considerable (I^2 = 96%).

Tesaglitazar

One RCT (2 comparisons) enrolling 590 patients reported this outcome [40]. The mean fasting insulin was significantly lower in patients treated with tesaglitizar plus metformin versus metformin alone (MD = -32.35 pmol/L 95% CI = -41.64, -23.05; p <0.001). Heterogeneity among pooled RCTs was not important (I^2 = 4%).

HOMA-B

Pioglitazone

One RCT enrolling 213 patients reported HOMA-B [39]. The mean HOMA-B was not significantly higher in patients treated with pioglitazone plus metformin versus metformin alone (MD = -4.87, 95% CI = -14.97, 5.23; p =0.34).

Rosiglitazone

Three RCTs (3 comparisons) enrolling 1274 patients reported HOMA-B [34, 45, 46]. The mean HOMA-B was significantly higher in patients treated with rosiglitazone plus metformin versus metformin alone (MD = 8.84, 95% CI = 7.03, 10.64; p <0.001). The heterogeneity among pooled RCTs was not important (I^2 = 0%).

hsCRP

Pioglitazone

Three RCTs enrolling 286 patients reported hsCRP [24, 25, 47]. The mean hsCRP was significantly lower in patients treated with pioglitazone plus metformin versus metformin alone (MD = -0.64 mg/L, 95% CI = -1.19, -0.08; p=0.02). Heterogeneity among pooled RCTs was moderate (I^2 = 41%).

Rosiglitazone

Five RCTs (5 comparisons) enrolling 1664 patients reported hsCRP [22, 34, 42, 44, 46]. The mean hsCRP was significantly lower in patients treated with rosiglitazone plus metformin versus metformin alone (MD = -0.61 mg/L, 95% CI = -0.92, -0.32; p=0.0001). Heterogeneity among pooled RCTs was substantial (I^2 = 88%).

Tesaglitazar

One RCT (2 comparisons) enrolling 590 patients reported this outcome [40]. There was no significant difference in mean hsCRP in patients treated with tesaglitizar plus metformin versus metformin alone (MD = -0.76 mg/L 95% CI = -1.84, 0.33; p=0.17). The heterogeneity among pooled RCTs was not important (I^2 = 0%).

Total cholesterol

Pioglitazone

Six RCTs enrolling 1238 patients reported total cholesterol [24, 25, 37, 39, 43, 47]. The mean total cholesterol was significantly higher in patients treated with pioglitazone plus metformin versus metformin alone (MD = 7.00 mg/dl, 95% CI = 6.53, 7.47; p <0.001). The heterogeneity among pooled RCTs was not important (I^2 = 0%).

Rosiglitazone

Eleven RCTs (13 comparisons) enrolling 3779 patients reported total cholesterol [22, 23, 34, 35, 38, 41, 42, 44-46, 48]. The mean total cholesterol was significantly higher in patients treated with rosiglitazone plus metformin versus metformin alone (MD = 17.53 mg/dl, 95% CI = 13.05, 22.00; p <0.001). Heterogeneity among pooled RCTs was considerable (I^2 = 94%).

Tesaglitazar

One RCT (2 comparisons) enrolling 590 patients reported this outcome [40]. There was no significant difference in mean total cholesterol in patients treated with tesaglitizar plus metformin versus metformin alone (MD = -1.02 mg/dl, 95% CI = -21.10, 19.06; p = 0.92). Heterogeneity among pooled RCTs was substantial (I^2 = 89%).

High-density lipoprotein cholesterol

Pioglitazone

Six RCTs enrolling 1238 patients reported HDL-cholesterol [24, 25, 37, 39, 43, 47]. The mean HDL-cholesterol was significantly higher in patients treated with pioglitazone plus metformin versus metformin alone (MD = 3.20 mg/dl, 95% CI = 1.81, 4.58; p <0.001). Heterogeneity among pooled RCTs was considerable (I^2 = 98%).

Rosiglitazone

Ten RCTs (12 comparisons) enrolling 3741 patients reported HDL-cholesterol [22, 34, 35, 38, 41, 42, 44-46, 48]. The mean HDL-cholesterol was significantly higher in patients treated with rosiglitazone plus metformin versus metformin alone (MD = 2.43 mg/dl, 95% CI = 1.13, 3.74; p =0.0003). Heterogeneity among pooled RCTs was substantial (I^2 = 84%).

Tesaglitazar

One RCT (2 comparisons) enrolling 590 patients reported this outcome [40]. The mean HDL-cholesterol was significantly higher in patients treated with tesaglitizar plus metformin versus metformin alone (MD = 4.25 mg/dl, 95% CI = 2.26, 6.24; p<0.001). The heterogeneity among pooled RCTs was not important (I^2 = 0%).

Low-density lipoprotein cholesterol

Pioglitazone

Six RCTs enrolling 1278 patients reported LDL-cholesterol [24, 25, 37, 39, 43, 47]. There was no significant difference in mean LDL-cholesterol in patients treated with pioglitazone plus metformin versus metformin alone (MD = 2.22 mg/dl, 95% CI = -3.15, 7.58; p=0.42). Heterogeneity among pooled RCTs was considerable (I^2 = 99%).

Rosiglitazone

Ten RCTs (12 comparisons) enrolling 3741 patients reported LDL-cholesterol [22, 34, 35, 38, 41, 42, 44-46, 48]. The mean LDL-cholesterol was significantly higher in patients treated with rosiglitazone plus metformin versus metformin alone (MD = 11.30 mg/dl, 95% CI = 8.46, 14.14; p <0.001). Heterogeneity among pooled RCTs was substantial (I^2 = 79%).

Tesaglitazar

One RCT (2 comparisons) enrolling 590 patients reported this outcome [40]. There was no significant difference in mean LDL-cholesterol in patients treated with tesaglitizar plus metformin versus metformin alone (MD = -1.36 mg/dl, 95% CI = -7.80, 5.08; p=0.68). Heterogeneity among pooled RCTs was not important (I^2 = 27%).

Triglycerides

Pioglitazone

Six RCTs enrolling 1238 patients reported triglycerides [24, 25, 37, 39, 43, 47]. There was no significant difference in mean triglycerides in patients treated with pioglitazone plus metformin versus metformin alone (MD = -20.87 mg/dl, 95% CI = -51.14, 9.39; p=0.18). Heterogeneity among pooled RCTs was considerable (I^2 = 98%).

Rosiglitazone

Eleven RCTs (13 comparisons) enrolling 3779 patients reported triglycerides [22, 23, 34, 35, 38, 41, 42, 44-46, 48]. There was no significant difference in mean triglycerides in patients treated with rosiglitazone plus metformin versus metformin alone (MD = 4.42 mg/dl, 95% CI = -8.84, 17.68; p=0.51). Heterogeneity among pooled RCTs was considerable (I^2 = 98%).

Tesaglitazar

One RCT (2 comparisons) enrolling 590 patients reported this outcome [40]. The mean triglycerides were significantly lower in patients treated with tesaglitizar plus metformin versus metformin alone (MD = -71.49 mg/dl, 95% CI = -115.84, -27.13; p=0.002). Heterogeneity among pooled RCTs was substantial (I^2 = 88%).

Systolic blood pressure

Pioglitazone

Two RCTs enrolling 249 patients reported systolic blood pressure (BP) [39, 47]. The mean systolic BP was significantly lower in patients treated with pioglitazone plus metformin versus metformin alone (MD = -3.99 mmHg, 95% CI = -7.26, -0.73; p=0.02). The heterogeneity among pooled RCTs was not important (I^2 = 0%).

Rosiglitazone

Four RCTs enrolling 804 patients reported systolic BP [22, 23, 42, 46]. The mean systolic BP was significantly lower in patients treated with rosiglitazone plus metformin versus metformin alone (MD = -3.06 mmHg, 95% CI = -5.22, -0.90; p =0.005). Heterogeneity among pooled RCTs was moderate (I^2 = 45%).

Diastolic blood pressure

Pioglitazone

Two RCTs enrolling 249 patients reported diastolic blood pressure (BP) [39, 47]. There was no significant difference in mean diastolic BP in patients treated with pioglitazone plus metformin

versus metformin alone (MD = -1.56 mmHg, 95% CI = -4.04, 0.93; p=0.22). Heterogeneity among pooled RCTs was not important (I^2 = 17%).

Rosiglitazone

Four RCTs enrolling 804 patients reported diastolic BP [22, 23, 42, 46]. The mean diastolic BP was significantly lower in patients treated with rosiglitazone plus metformin versus metformin alone (MD = -3.12 mmHg, 95% CI = -5.92, -0.32; p =0.03). Heterogeneity among pooled RCTs was substantial (I^2 = 86%).

Any adverse events

Pioglitazone

Five RCTs enrolling 1161 patients reported adverse events [24, 25, 33, 39, 43]. The risk of adverse events in patients treated with metformin plus PPAR agonist compared to patients treated with metformin alone was not significant (RR = 1.05, 95% CI = 0.96, 1.16; p = 0.29). Heterogeneity among pooled RCTs was not important (I^2 = 6%).

Rosiglitazone

Seven RCTs (9 comparisons) enrolling 3095 patients reported adverse events [34, 35, 38, 41, 45, 46, 48]. The risk of adverse events in patients treated with metformin plus PPAR agonist compared to patients treated with metformin alone was not significant (RR = 1.03, 95% CI = 0.96, 1.11; p = 0.44). Heterogeneity among pooled RCTs was not important (I^2 = 20%).

Tesaglitazar

One RCT (2 comparisons) enrolling 585 patients reported this outcome [40]. The risk of adverse events in patients treated with metformin plus PPAR agonist compared to patients treated with

metformin alone was not significant (RR = 0.92, 95% CI = 0.79, 1.07; p = 0.27). The heterogeneity among pooled RCTs was not important (I^2 = 0%).

Gastrointestinal intolerance

Pioglitazone

Two RCTs enrolling 604 patients reported gastrointestinal adverse events [39, 43]. Patients treated with metformin plus PPAR agonist had a significantly lower mean gastrointestinal adverse events compared to patients treated with metformin alone (RR = 0.61, 95% CI = 0.42, 0.89; p =0.01). The heterogeneity among pooled RCTs was not important (I^2 = 0%).

Rosiglitazone

Six RCTs enrolling 2894 patients reported gastrointestinal adverse events [34, 35, 44-46, 48]. Patients treated with metformin plus PPAR agonist had a significantly lower risk of gastrointestinal adverse events compared to patients treated with metformin alone (RR = 0.83, 95% CI = 0.74, 0.93; p =0.001). The heterogeneity among pooled RCTs was not important (I^2 = 7%).

Tesaglitazar

One RCT (2 comparisons) enrolling 585 patients reported this outcome [40]. The risk of gastrointestinal adverse events in patients treated with metformin plus PPAR agonist compared to patients treated with metformin alone was not significant (RR = 0.79, 95% CI = 0.35, 1.81; p = 0.58). The heterogeneity among pooled RCTs was not important (I^2 = 0%).

Supplementary Material: Search strategy

PubMed search strategy

**Search number**	**Search Query**	**Search item**
1	"diabetes mellitus, type 2"[MeSH Terms]	
2	"MODY"[Title/Abstract] OR "NIDDM"[Title/Abstract] OR "t2d"[Title/Abstract]	
3	"non insulin depend*"[Title/Abstract] OR "noninsulin depend*"[Title/Abstract] OR "noninsulin-depend*"[Title/Abstract] OR "non insulin-depend*"[Title/Abstract]	
4	"late onset diabet*"[Title/Abstract] OR "adult onset diabet*"[Title/Abstract] OR ("matur*"[All Fields] AND "onset diabet*"[Title/Abstract]) OR "slow onset diabet*"[Title/Abstract] OR ("stabl*"[All Fields] AND "onset diabet*"[Title/Abstract])	
5	"type 2 diabet*"[Title/Abstract] OR "type ii diabet*"[Title/Abstract] OR "typ 2 diabet*"[Title/Abstract] OR "typ ii diabet*"[Title/Abstract]	
6	**1 OR 2 OR 3 OR 4 OR 5**	**Diabetes search**
7	"metformin"[MeSH Terms]	
8	"metformin*"[Title/Abstract]	
9	**7 OR 8**	**Metformin search**
10	**6 AND 9**	**Diabetes + Metformin**
11	"peroxisome proliferator activated receptors/agonists"[MeSH Terms]	
12	"peroxisome proliferator activated receptor agonist*"[Title/Abstract] OR "ppar agonist*"[Title/Abstract] OR "ppar alpha agonist*"[Title/Abstract] OR "ppar gamma agonist*"[Title/Abstract]	
13	"saroglitazar"[Supplementary Concept]	
14	"saroglitazar"[Title/Abstract]	
15	"chiglitazar"[Supplementary Concept]	
16	"chiglitazar"[Title/Abstract]	
17	"aleglitazar"[Supplementary Concept]	
18	"aleglitazar"[Title/Abstract]	
19	"naveglitazar"[Supplementary Concept]	
20	"naveglitazar"[Title/Abstract]	
21	"Rosiglitazone"[MeSH Terms]	
22	"Rosiglitazone"[Title/Abstract]	
23	"rosiglitazone-metformin combination"[Supplementary Concept]	
24	"Pioglitazone"[MeSH Terms]	
25	"Pioglitazone"[Title/Abstract]	
26	"Lobeglitazone"[Title/Abstract]	
27	"ragaglitazar"[Supplementary Concept]	

28	"ragaglitazar"[Title/Abstract]	
29	"Imiglitazar"[Title/Abstract]	
30	"tesaglitazar"[Supplementary Concept]	
31	"tesaglitazar"[Title/Abstract]	
32	"peliglitazar"[Supplementary Concept]	
33	"peliglitazar"[Title/Abstract]	
34	"farglitazar"[Supplementary Concept]	
35	"farglitazar"[Title/Abstract]	
36	"sipoglitazar"[Supplementary Concept]	
37	"sipoglitazar"[Title/Abstract]	
38	"indeglitazar"[Title/Abstract]	
39	"muraglitazar"[Supplementary Concept]	
40	"muraglitazar"[Title/Abstract]	
41	"Troglitazone"[MeSH Terms]	
42	"Troglitazone"[Title/Abstract]	
43	"rivoglitazone"[Supplementary Concept]	
44	"rivoglitazone"[Title/Abstract]	
45	"balaglitazone"[Supplementary Concept]	
46	"balaglitazone"[Title/Abstract]	
47	"ciglitazone"[Supplementary Concept]	
48	"ciglitazone"[Title/Abstract]	
49	"netoglitazone"[Supplementary Concept]	
50	"netoglitazone"[Title/Abstract]	
51	"isaglitazone"[Title/Abstract]	
52	"BM 13.1258"[Supplementary Concept]	
53	"edaglitazone"[Title/Abstract]	
54	"darglitazone"[Supplementary Concept]	
55	"darglitazone"[Title/Abstract]	
56	11 OR 12 OR 13 OR 14 OR 15 OR 16 OR 17 OR 18 OR 19 OR 20 OR 21 OR 22 OR 23 OR 24 OR 25 OR 26 OR 27 OR 28 OR 29 OR 30 OR 31 OR 32 OR 33 OR 34 OR 35 OR 36 OR 37 OR 38 OR 39 OR 40 OR 41 OR 42 OR 43 OR 44 OR 45 OR 46 OR 47 OR 48 OR 49 OR 50 OR 51 OR 52 OR 53 OR 54 OR 55	**PPAR agonist**
57	10 AND 56	**Diabetes + Metformin + PPAR agonist**
58	("randomized controlled trial"[Publication Type] OR "controlled clinical trial"[Publication Type] OR "randomized"[Title/Abstract] OR "placebo"[Title/Abstract] OR "drug therapy"[MeSH Subheading] OR "randomly"[Title/Abstract] OR "trial"[Title/Abstract] OR "groups"[Title/Abstract]) NOT ("animals"[MeSH Terms] NOT "humans"[MeSH Terms])	**RCT filter**
59	57 AND 58	**Final search**

EMBASE search strategy

Search number	Search Query	Search item
1	'non insulin dependent diabetes mellitus'/exp	
2	'non insulin dependent diabetes mellitus':ab,ti	
3	'niddm':ab,ti	
4	'non insulin depend*':ab,ti OR 'noninsulin depend*':ab,ti	
5	'late onset diabet*':ab,ti OR 'adult onset diabet*':ab,ti OR ('matur*':ab,ti AND 'onset diabet*':ab,ti) OR 'slow onset diabet*':ab,ti OR ('stabl*':ab,ti AND 'onset diabet*':ab,ti)	
6	'ltype 2 diabet*':ab,ti OR 'type ii diabet*':ab,ti OR 'typ 2 diabet*':ab,ti OR 'typ ii diabet':ab,ti	
7	**#1 OR #2 OR #3 OR #4 OR #5 OR #6**	**Diabetes search**
8	**'metformin'/exp OR 'metformin'**	**Metformin search**
9	**#7 AND #8**	**Diabetes + Metformin**
10	'peroxisome proliferator activated receptor agonist'/de OR 'peroxisome proliferator activated receptor agonist':ab,ti	
11	'peroxisome proliferator activated receptor agonist'/de OR 'peroxisome proliferator activated receptor agonist*':ab,ti OR 'ppar agonist*':ab,ti	
12	'saroglitazar'/de OR 'saroglitazar':ab,ti	
13	'chiglitazar'/de OR 'chiglitazar':ab,ti	
14	'aleglitazar'/de OR 'aleglitazar':ab,ti	
15	'naveglitazar'/de OR 'naveglitazar':ab,ti	
16	'rosiglitazone'/de OR 'rosiglitazone':ab,ti	
17	'pioglitazone'/de OR 'pioglitazone':ab,ti	
18	'lobeglitazone'/de OR 'lobeglitazone':ab,ti	
19	'ragaglitazar'/de OR 'ragaglitazar':ab,ti	
20	'imiglitazar'/de OR 'imiglitazar':ab,ti	
21	'tesaglitazar'/de OR 'tesaglitazar':ab,ti	
22	'peliglitazar'/de OR 'peliglitazar':ab,ti	
23	'farglitazar'/de OR 'farglitazar':ab,ti	
24	'sipoglitazar'/de OR 'sipoglitazar':ab,ti	
25	'indeglitazar'/de OR 'indeglitazar':ab,ti	
26	'muraglitazar'/de OR 'muraglitazar':ab,ti	
27	'troglitazone'/de OR 'troglitazone':ab,ti	
28	'rivoglitazone'/de OR 'rivoglitazone':ab,ti	
29	'balaglitazone'/de OR 'balaglitazone':ab,ti	
30	'ciglitazone'/de OR 'ciglitazone':ab,ti	
31	'netoglitazone'/de OR 'netoglitazone':ab,ti	
32	'isaglitazone' OR 'isaglitazone':ab,ti	

33	'edaglitazone'/de OR 'edaglitazone':ab,ti	
34	'darglitazone'/de OR 'darglitazone':ab,ti	
35	**#10 OR #11 OR #12 OR #13 OR #14 OR #15 OR #16 OR #17 OR #18 OR #19 OR #20 OR #21 OR #22 OR #23 OR #24 OR #25 OR #26 OR #27 OR #28 OR #29 OR #30 OR #31 OR #32 OR #33 OR #34**	**PPAR agonist**
36	**#9 AND #35**	**Diabetes + Metformin + PPAR agonist**
37	'randomized controlled trial'/de OR 'controlled clinical trial'/de OR 'random$':ti,ab OR 'randomization'/de OR 'intermethod comparison'/de OR 'placebo':ti,ab	
38	'compare':ti OR 'compared':ti OR 'comparison':ti	
39	(evaluated:ab OR evaluate:ab OR evaluating:ab OR assessed:ab OR assess:ab) AND (compare:ab OR compared:ab OR comparing:ab OR comparison:ab)	
40	'double blind procedure'/de OR 'parallel group*':ti,ab	
41	(double:ti,ab OR single:ti,ab OR doubly:ti,ab OR singly:ti,ab) AND (blind:ti,ab OR blinded:ti,ab OR blindly:ti,ab)	
42	open:ti,ab AND label:ti,ab	
43	(crossover:ti,ab OR cross:ti,ab) AND over:ti,ab	
44	(assign*:ti,ab OR match:ti,ab OR matched:ti,ab OR allocation:ti,ab) AND (alternate:ti,ab OR group*:ti,ab OR intervention*:ti,ab OR patient*:ti,ab OR subject*:ti,ab OR participant*:ti,ab)	
45	assigned:ti,ab OR allocated:ti,ab	
46	controlled:ti,ab AND (study:ti,ab OR design:ti,ab OR trial:ti,ab)	
47	volunteer:ti,ab OR volunteers:ti,ab	
48	#37 OR #38 OR #39 OR #40 OR #41 OR #42 OR #43 OR #44 OR #45 OR #46 OR #47	
49	(rat:ti OR rats:ti OR mouse:ti OR mice:ti OR swine:ti OR porcine:ti OR murine:ti OR sheep:ti OR lambs:ti OR pigs:ti OR piglets:ti OR rabbit:ti OR rabbits:ti OR cat:ti OR cats:ti OR dog:ti OR dogs:ti OR cattle:ti OR bovine:ti OR monkey:ti OR monkeys:ti OR trout:ti OR marmoset:ti) AND 'animal experiment'/de	
50	'animal experiment'/de NOT ('human'/de OR 'human experiment'/de)	
51	#49 OR #50	
52	**#48 NOT #51**	**RCT filter**
53	**#36 AND #52**	**Final search**

Supplement Figure 1. Risk of bias summary

	Random sequence generation (selection bias)	Allocation concealment (selection bias)	Blinding of participants and personnel (performance bias)	Blinding of outcome assessment (detection bias)	Incomplete outcome data (attrition bias)	Selective reporting (reporting bias)	Other bias
Bailey 2005	+	?	+	+	−	+	+
Borges 2011	?	?	+	+	−	+	+
Derosa 2009	+	+	+	+	−	+	+
Einhorn 2000	?	?	+	+	−	+	+
Fonseca 2000 (high dose)	+	+	+	+	−	+	+
Fonseca 2000 (low dose)	+	+	+	+	−	+	+
Genovese 2013	?	?	+	+	+	+	+
Goke 2007 (high dose)	?	?	+	+	−	+	+
Goke 2007 (low dose)	?	?	+	+	−	+	+
Gomez-Perez 2002 (high dose)	?	?	−	?	−	+	+
Gomez-Perez 2002 (low dose)	?	?	−	?	−	+	+
Hanefeld 2011	?	?	+	+	−	+	+
Kadoglou 2010	+	+	?	?	−	+	+
Kadoglou 2011	?	?	−	−	−	+	+
Kaku 2009	?	?	+	+	−	+	+
Negro 2005	?	?	+	+	?	+	+
Perez 2009	?	?	+	+	−	−	+
Rosenstock 2006	?	?	+	+	−	+	+
Scott 2008	?	?	+	?	−	+	+
Stewart 2006	?	?	+	+	−	+	+
Takeda 2008	?	?	+	+	−	+	+
Wang 2006	+	?	+	+	?	−	+
Weissman 2005	?	?	+	+	−	+	+

Supplement Figure 2. HOMA-IR

Study or Subgroup	PPAR agonist plus metformin Mean	SD	Total	Metformin alone Mean	SD	Total	Weight	Mean Difference IV, Random, 95% CI
**Pioglitazone**								
Genovese 2013	2.88	1.95	110	4.68	3.63	103	14.7%	-1.80 [-2.59, -1.01]
Wang 2006	2.93	2.99	24	4.1	2.25	12	10.2%	-1.17 [-2.92, 0.58]
**Subtotal (95% CI)**			**134**			**115**	**24.9%**	**-1.69 [-2.41, -0.97]**
Heterogeneity: Tau² = 0.00; Chi² = 0.41, df = 1 (P = 0.52); I² = 0%								
Test for overall effect: Z = 4.61 (P < 0.00001)								
**Rosiglitazone**								
Kadoglou 2010	3.28	1.37	50	3.98	1.34	50	15.7%	-0.70 [-1.23, -0.17]
Kadoglou 2011	3.17	1.41	70	2.91	1.19	70	16.0%	0.26 [-0.17, 0.69]
Kaku 2009	2.55	2.38	83	3.37	2.69	86	14.9%	-0.82 [-1.59, -0.05]
Negro 2005	5.2	1.05	19	7.9	1.1	19	15.2%	-2.70 [-3.38, -2.02]
Scott 2008	3.7	3.1	87	5.8	4.4	92	13.3%	-2.10 [-3.21, -0.99]
**Subtotal (95% CI)**			**309**			**317**	**75.1%**	**-1.17 [-2.27, -0.08]**
Heterogeneity: Tau² = 1.42; Chi² = 57.95, df = 4 (P < 0.00001); I² = 93%								
Test for overall effect: Z = 2.11 (P = 0.04)								
**Total (95% CI)**			**443**			**432**	**100.0%**	**-1.26 [-2.16, -0.37]**
Heterogeneity: Tau² = 1.25; Chi² = 64.32, df = 6 (P < 0.00001); I² = 91%								
Test for overall effect: Z = 2.77 (P = 0.006)								
Test for subgroup differences: Chi² = 0.60, df = 1 (P = 0.44), I² = 0%								

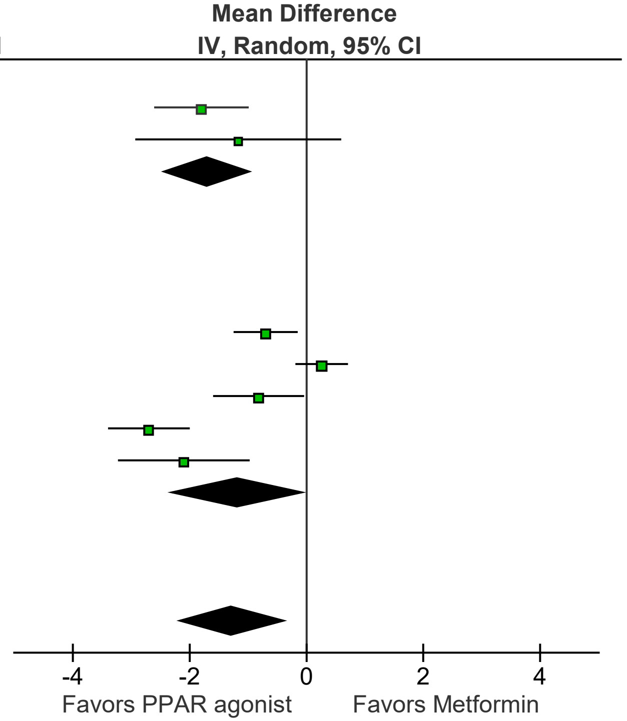

Supplement Figure 3. Fasting insulin

	PPAR agonist plus metformin			Metformin alone				Mean Difference
**Study or Subgroup**	**Mean**	**SD**	**Total**	**Mean**	**SD**	**Total**	**Weight**	**IV, Random, 95% CI**
**Pioglitazone**								
Derosa 2009	123.61	32.64	69	159.03	40.28	67	6.5%	-35.42 [-47.76, -23.08]
Genovese 2013	61.19	30.35	110	86.26	55.7	103	6.5%	-25.07 [-37.23, -12.91]
Hanefeld 2011	42.36	29.51	39	86.32	72.78	42	5.1%	-43.96 [-67.84, -20.08]
Kaku 2009	51.81	33.06	83	58.75	35.42	86	6.7%	-6.94 [-17.27, 3.39]
Wang 2006	71.53	65.28	24	98.62	52.78	12	3.3%	-27.09 [-66.76, 12.58]
**Subtotal (95% CI)**			**325**			**310**	**28.2%**	**-26.17 [-40.46, -11.89]**
Heterogeneity: Tau² = 178.13; Chi² = 16.26, df = 4 (P = 0.003); I² = 75%								
Test for overall effect: Z = 3.59 (P = 0.0003)								
**Rosiglitazone**								
Bailey 2005	58.9	54.8	289	72.1	55.4	280	6.9%	-13.20 [-22.26, -4.14]
Fonseca 2000 (high dose)	105.66	30.35	113	107.51	55.7	58	6.2%	-1.85 [-17.24, 13.54]
Fonseca 2000 (low dose)	111.57	30.35	119	107.51	55.7	58	6.2%	4.06 [-11.28, 19.40]
Kadoglou 2010	63.4	28.82	50	69.58	27.08	50	6.7%	-6.18 [-17.14, 4.78]
Kadoglou 2011	62.99	44.79	70	72.92	52.85	70	6.1%	-9.93 [-26.16, 6.30]
Negro 2005	86.81	14.58	19	112.5	13.89	19	6.9%	-25.69 [-34.74, -16.64]
Rosenstock 2006	83.53	8.6	155	125.78	11.7	154	7.3%	-42.25 [-44.54, -39.96]
Scott 2008	77.78	61.11	87	95.83	57.64	92	5.9%	-18.05 [-35.47, -0.63]
Stewart 2006	65.2	47.7	254	70.9	49.4	272	6.9%	-5.70 [-14.00, 2.60]
**Subtotal (95% CI)**			**1156**			**1053**	**58.9%**	**-13.59 [-28.06, 0.89]**
Heterogeneity: Tau² = 451.74; Chi² = 194.56, df = 8 (P < 0.00001); I² = 96%								
Test for overall effect: Z = 1.84 (P = 0.07)								
**Tesaglitazar**								
Goke 2007 (high dose)	35.2	54.8	196	72.6	55.4	100	6.4%	-37.40 [-50.70, -24.10]
Goke 2007 (low dose)	44.7	43	194	72.6	55.4	100	6.5%	-27.90 [-40.33, -15.47]
**Subtotal (95% CI)**			**390**			**200**	**12.9%**	**-32.35 [-41.64, -23.05]**
Heterogeneity: Tau² = 2.01; Chi² = 1.05, df = 1 (P = 0.31); I² = 4%								
Test for overall effect: Z = 6.82 (P < 0.00001)								
**Total (95% CI)**			**1871**			**1563**	**100.0%**	**-19.83 [-29.54, -10.13]**
Heterogeneity: Tau² = 336.12; Chi² = 224.87, df = 15 (P < 0.00001); I² = 93%								
Test for overall effect: Z = 4.00 (P < 0.0001)								
Test for subgroup differences: Chi² = 4.58, df = 2 (P = 0.10), I² = 56.3%								

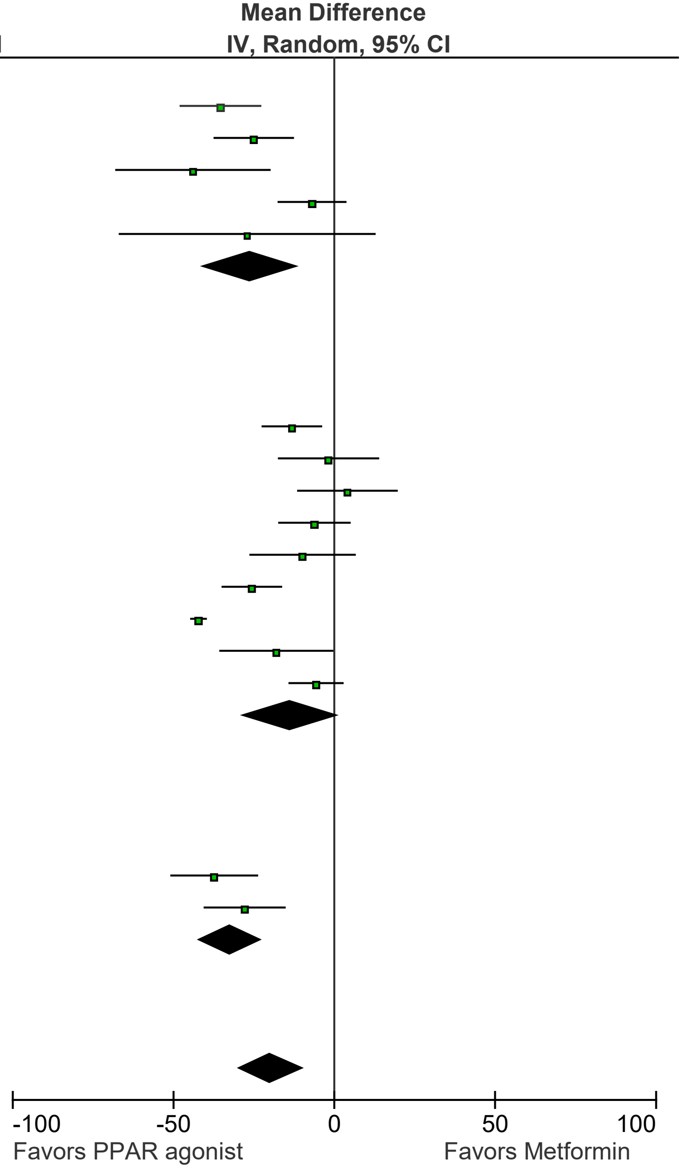

Supplement Figure 4. HOMA-B

Study or Subgroup	PPAR agonist plus metformin Mean	SD	Total	Metformin alone Mean	SD	Total	Weight	Mean Difference IV, Random, 95% CI
**Pioglitazone**								
Genovese 2013	52.42	28.02	110	57.29	44.71	103	11.9%	-4.87 [-14.97, 5.23]
**Subtotal (95% CI)**			**110**			**103**	**11.9%**	**-4.87 [-14.97, 5.23]**
Heterogeneity: Not applicable								
Test for overall effect: Z = 0.95 (P = 0.34)								
**Rosiglitazone**								
Bailey 2005	66.53	15.69	289	56.89	14.9	280	41.8%	9.64 [7.13, 12.15]
Scott 2008	71	74.2	87	55.4	32.5	92	5.0%	15.60 [-1.35, 32.55]
Stewart 2006	84.1	15.69	254	76.3	14.9	272	41.2%	7.80 [5.18, 10.42]
**Subtotal (95% CI)**			**630**			**644**	**88.1%**	**8.84 [7.03, 10.64]**
Heterogeneity: Tau² = 0.00; Chi² = 1.61, df = 2 (P = 0.45); I² = 0%								
Test for overall effect: Z = 9.60 (P < 0.00001)								
**Total (95% CI)**			**740**			**747**	**100.0%**	**7.45 [3.45, 11.45]**
Heterogeneity: Tau² = 8.31; Chi² = 8.46, df = 3 (P = 0.04); I² = 65%								
Test for overall effect: Z = 3.65 (P = 0.0003)								
Test for subgroup differences: Chi² = 6.86, df = 1 (P = 0.009), I² = 85.4%								

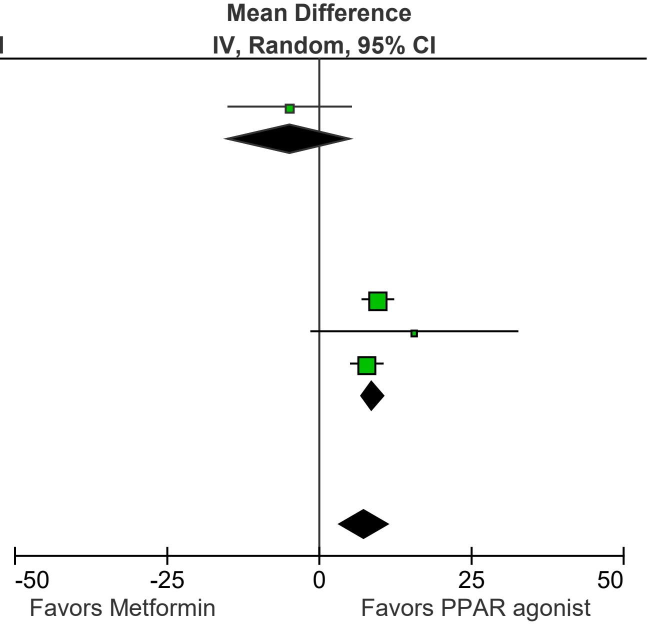

Supplement Figure 5. hsCRP

Study or Subgroup	PPAR agonist plus metformin Mean	SD	Total	Metformin alone Mean	SD	Total	Weight	Mean Difference IV, Random, 95% CI
**Pioglitazone**								
Hanefeld 2011	1.78	1.06	39	2.99	2.42	42	6.2%	-1.21 [-2.01, -0.41]
Kaku 2009	0.8	1.6	83	1.15	1.3	86	11.3%	-0.35 [-0.79, 0.09]
Wang 2006	1.21	1.87	24	1.75	1.52	12	3.7%	-0.54 [-1.68, 0.60]
**Subtotal (95% CI)**			**146**			**140**	**21.2%**	**-0.64 [-1.19, -0.08]**
Heterogeneity: Tau² = 0.10; Chi² = 3.38, df = 2 (P = 0.18); I² = 41%								
Test for overall effect: Z = 2.26 (P = 0.02)								
**Rosiglitazone**								
Bailey 2005	2	1.1	289	2.8	1	280	16.3%	-0.80 [-0.97, -0.63]
Kadoglou 2010	1.45	0.71	50	2.15	0.92	50	13.6%	-0.70 [-1.02, -0.38]
Kadoglou 2011	1.5	0.9	70	2.6	1	70	13.7%	-1.10 [-1.42, -0.78]
Rosenstock 2006	2.8	1.2	155	3	1.2	154	14.6%	-0.20 [-0.47, 0.07]
Stewart 2006	2.1	1.1	254	2.4	1.1	272	16.0%	-0.30 [-0.49, -0.11]
**Subtotal (95% CI)**			**818**			**826**	**74.2%**	**-0.61 [-0.92, -0.30]**
Heterogeneity: Tau² = 0.11; Chi² = 33.47, df = 4 (P < 0.00001); I² = 88%								
Test for overall effect: Z = 3.87 (P = 0.0001)								
**Tesaglitazar**								
Goke 2007 (high dose)	3.02	6.36	196	4.03	6.24	100	2.3%	-1.01 [-2.52, 0.50]
Goke 2007 (low dose)	3.54	6.84	194	4.03	6.24	100	2.2%	-0.49 [-2.05, 1.07]
**Subtotal (95% CI)**			**390**			**200**	**4.5%**	**-0.76 [-1.84, 0.33]**
Heterogeneity: Tau² = 0.00; Chi² = 0.22, df = 1 (P = 0.64); I² = 0%								
Test for overall effect: Z = 1.37 (P = 0.17)								
**Total (95% CI)**			**1354**			**1166**	**100.0%**	**-0.62 [-0.87, -0.37]**
Heterogeneity: Tau² = 0.09; Chi² = 37.21, df = 9 (P < 0.0001); I² = 76%								
Test for overall effect: Z = 4.93 (P < 0.00001)								
Test for subgroup differences: Chi² = 0.07, df = 2 (P = 0.97), I² = 0%								

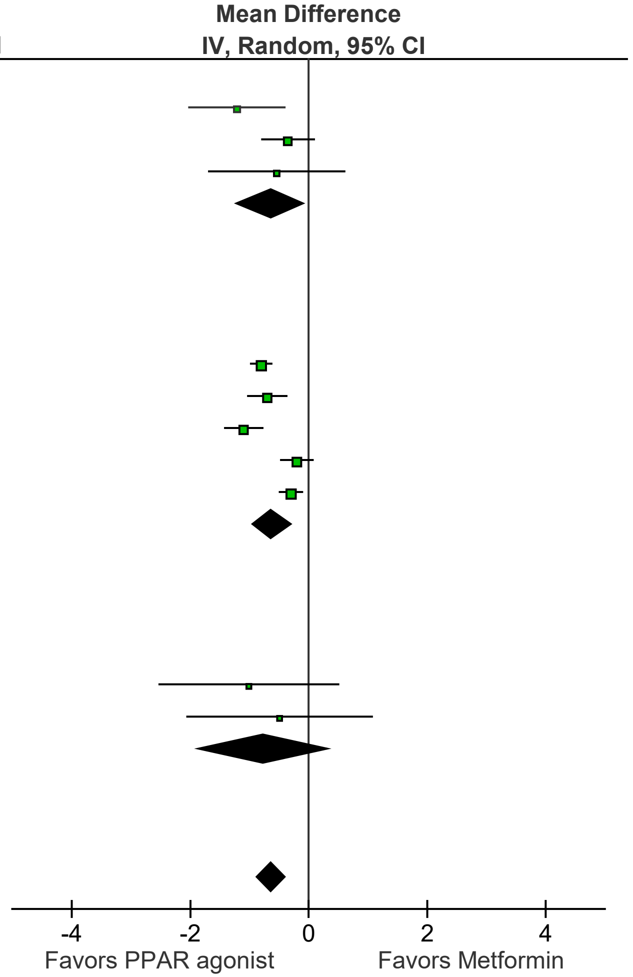

Supplement Figure 6. Total cholesterol

Study or Subgroup	PPAR agonist plus metformin Mean	SD	Total	Metformin alone Mean	SD	Total	Weight	Mean Difference IV, Random, 95% CI
**Pioglitazone**								
Einhorn 2000	221.5248	4.12	168	214.2309	4.28	160	7.7%	7.29 [6.38, 8.20]
Genovese 2013	187.8	33.7	110	176.2	32	103	5.0%	11.60 [2.78, 20.42]
Hanefeld 2011	188.3229	33.2562	39	186.3894	34.803	42	3.1%	1.93 [-12.89, 16.76]
Kaku 2009	207.3	23.5	83	202.5	22.2	86	5.8%	4.80 [-2.10, 11.70]
Perez 2009	197.5723	2.85	201	190.6688	2.79	210	7.7%	6.90 [6.36, 7.45]
Wang 2006	188	67.5	24	201.9	37.2	12	0.8%	-13.90 [-48.14, 20.34]
**Subtotal (95% CI)**			**625**			**613**	**30.0%**	**7.00 [6.53, 7.47]**
Heterogeneity: Tau² = 0.00; Chi² = 3.84, df = 5 (P = 0.57); I² = 0%								
Test for overall effect: Z = 29.45 (P < 0.00001)								
**Rosiglitazone**								
Bailey 2005	220.8	17.44	289	200.78	15.41	280	7.3%	20.02 [17.32, 22.72]
Borges 2011	194.33	9.26	348	184.73	9.63	340	7.6%	9.60 [8.19, 11.01]
Fonseca 2000 (high dose)	232.4067	54.5247	113	212.685	39.8301	58	3.2%	19.72 [5.36, 34.08]
Fonseca 2000 (low dose)	231.2466	40.6035	119	212.685	39.8301	58	3.7%	18.56 [5.98, 31.14]
Gomez-Perez 2002 (high dose)	220.4	39	40	204.2	32.7	20	2.2%	16.20 [-2.55, 34.95]
Gomez-Perez 2002 (low dose)	209.2	35	37	204.2	32.7	19	2.3%	5.00 [-13.53, 23.53]
Kadoglou 2010	213.3	55.1	50	201.3	56.4	50	1.8%	12.00 [-9.86, 33.86]
Kadoglou 2011	215	58	70	196	51	70	2.4%	19.00 [0.91, 37.09]
Negro 2005	190	21.1	19	165.3	19.6	19	3.6%	24.70 [11.75, 37.65]
Rosenstock 2006	196.12	7.58	155	183.42	7.77	154	7.5%	12.70 [10.99, 14.41]
Scott 2008	206.6	49.1	87	190.4	33.6	92	3.7%	16.20 [3.81, 28.59]
Stewart 2006	217.119	8.586	254	197.5	9.04	272	7.6%	19.62 [18.11, 21.13]
Weissman 2005	215.7	17.18	382	188.77	14.55	384	7.4%	26.93 [24.67, 29.19]
**Subtotal (95% CI)**			**1963**			**1816**	**60.3%**	**17.53 [13.05, 22.00]**
Heterogeneity: Tau² = 41.98; Chi² = 217.82, df = 12 (P < 0.00001); I² = 94%								
Test for overall effect: Z = 7.67 (P < 0.00001)								
**Tesaglitazar**								
Goke 2007 (high dose)	176.3352	35.5764	196	187.5495	38.67	100	4.9%	-11.21 [-20.28, -2.15]
Goke 2007 (low dose)	196.8303	41.3769	194	187.5495	38.67	100	4.7%	9.28 [-0.28, 18.84]
**Subtotal (95% CI)**			**390**			**200**	**9.6%**	**-1.02 [-21.11, 19.06]**
Heterogeneity: Tau² = 187.43; Chi² = 9.30, df = 1 (P = 0.002); I² = 89%								
Test for overall effect: Z = 0.10 (P = 0.92)								
**Total (95% CI)**			**2978**			**2629**	**100.0%**	**12.36 [9.02, 15.70]**
Heterogeneity: Tau² = 37.63; Chi² = 620.48, df = 20 (P < 0.00001); I² = 97%								
Test for overall effect: Z = 7.26 (P < 0.00001)								
Test for subgroup differences: Chi² = 21.63, df = 2 (P < 0.0001), I² = 90.8%								

Mean Difference IV, Random, 95% CI

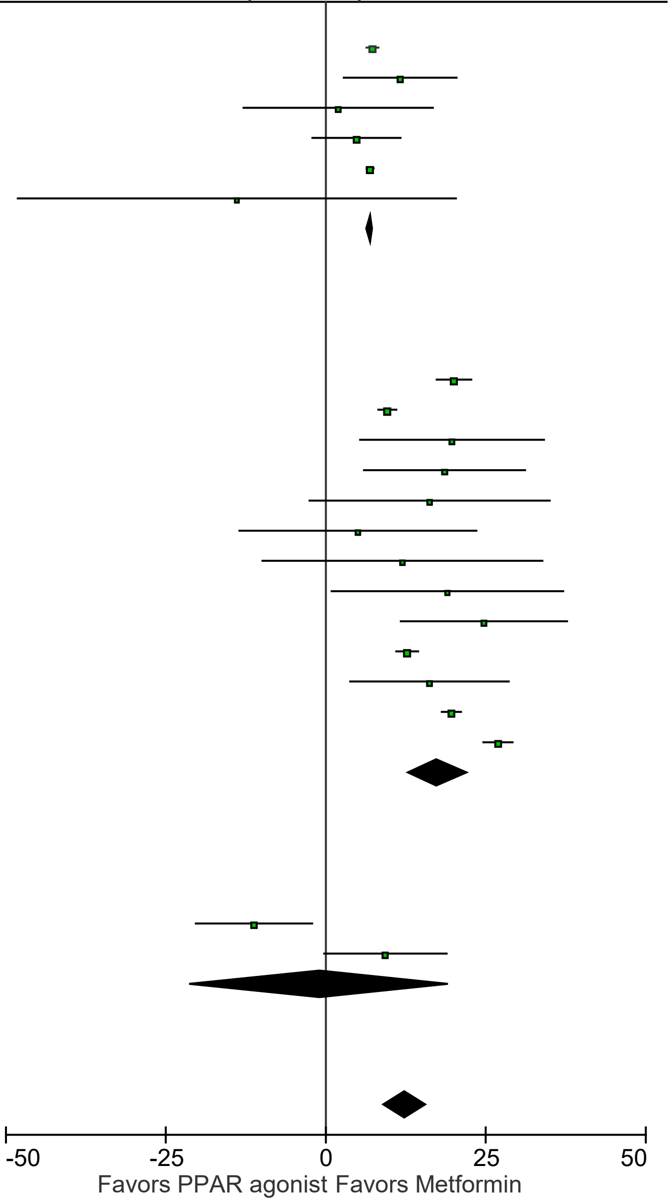

Supplement Figure 7. High-density lipoprotein-cholesterol (HDL-C)

Study or Subgroup	PPAR agonist plus metformin Mean	SD	Total	Metformin alone Mean	SD	Total	Weight	Mean Difference IV, Random, 95% CI
**Pioglitazone**								
Einhorn 2000	47.28	0.95	168	42.73	1	160	9.7%	4.55 [4.34, 4.76]
Genovese 2013	40.72	7.6	110	37.42	7.76	103	6.0%	3.30 [1.24, 5.36]
Hanefeld 2011	54.52	13.92	39	49.88	10.05	42	1.9%	4.64 [-0.68, 9.96]
Kaku 2009	56.5	8.1	83	54.5	8.5	86	5.1%	2.00 [-0.50, 4.50]
Perez 2009	47.74	0.76	201	45.09	0.75	210	9.7%	2.65 [2.50, 2.80]
Wang 2006	34.1	9.2	24	34	7.8	12	1.7%	0.10 [-5.65, 5.85]
**Subtotal (95% CI)**			**625**			**613**	**34.0%**	**3.20 [1.81, 4.58]**
Heterogeneity: Tau² = 1.74; Chi² = 212.60, df = 5 (P < 0.00001); I² = 98%								
Test for overall effect: Z = 4.52 (P < 0.00001)								
**Rosiglitazone**								
Bailey 2005	47.11	4.43	289	46.4	3.13	280	9.2%	0.71 [0.08, 1.34]
Borges 2011	48.1	10.07	348	46.83	9.63	340	7.4%	1.27 [-0.20, 2.74]
Fonseca 2000 (high dose)	52.59	16.24	113	46.4	11.21	58	2.7%	6.19 [2.03, 10.35]
Fonseca 2000 (low dose)	51.04	13.15	119	46.4	11.21	58	3.2%	4.64 [0.91, 8.37]
Gomez-Perez 2002 (high dose)	57.9	10.9	40	48.9	11.9	20	1.5%	9.00 [2.79, 15.21]
Gomez-Perez 2002 (low dose)	53.3	10	37	48.9	11.9	19	1.4%	4.40 [-1.85, 10.65]
Kadoglou 2010	45.9	8.9	50	47.3	12.2	50	2.7%	-1.40 [-5.59, 2.79]
Kadoglou 2011	47	11	70	47	12	70	3.1%	0.00 [-3.81, 3.81]
Rosenstock 2006	45.03	9.71	155	43.03	8.78	154	6.0%	2.00 [-0.06, 4.06]
Scott 2008	45.7	10.5	87	44.1	12.1	92	3.7%	1.60 [-1.71, 4.91]
Stewart 2006	49.79	10.61	254	47.19	9.53	272	6.8%	2.60 [0.87, 4.33]
Weissman 2005	49.11	4.36	382	45.3	3.64	384	9.3%	3.81 [3.24, 4.38]
**Subtotal (95% CI)**			**1944**			**1797**	**57.0%**	**2.43 [1.13, 3.74]**
Heterogeneity: Tau² = 3.14; Chi² = 67.97, df = 11 (P < 0.00001); I² = 84%								
Test for overall effect: Z = 3.65 (P = 0.0003)								
**Tesaglitazar**								
Goke 2007 (high dose)	52.2	11.60	196	48.34	11.6	100	4.5%	3.86 [1.07, 6.65]
Goke 2007 (low dose)	52.98	11.99	194	48.34	11.6	100	4.5%	4.64 [1.81, 7.47]
**Subtotal (95% CI)**			**390**			**200**	**9.0%**	**4.24 [2.26, 6.23]**
Heterogeneity: Tau² = 0.00; Chi² = 0.15, df = 1 (P = 0.70); I² = 0%								
Test for overall effect: Z = 4.18 (P < 0.0001)								
**Total (95% CI)**			**2959**			**2610**	**100.0%**	**2.81 [2.00, 3.62]**
Heterogeneity: Tau² = 1.76; Chi² = 302.02, df = 19 (P < 0.00001); I² = 94%								
Test for overall effect: Z = 6.78 (P < 0.00001)								
Test for subgroup differences: Chi² = 2.29, df = 2 (P = 0.32), I² = 12.6%								

Mean Difference
IV, Random, 95% CI

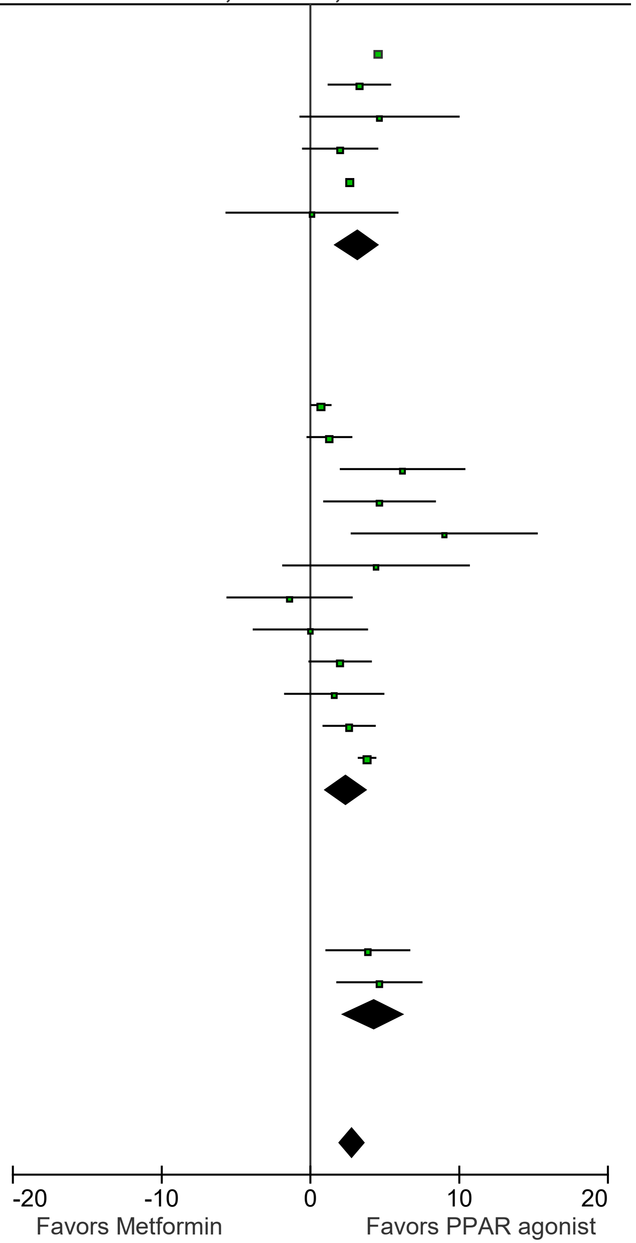

Supplement Figure 8. Low-density lipoprotein-cholesterol (LDL-C)

	PPAR agonist plus metformin			Metformin alone				Mean Difference
**Study or Subgroup**	**Mean**	**SD**	**Total**	**Mean**	**SD**	**Total**	**Weight**	**IV, Random, 95% CI**
**Pioglitazone**								
Einhorn 2000	128.49	3.07	168	132.04	3.15	160	7.2%	-3.55 [-4.22, -2.88]
Genovese 2013	115.4	29.4	110	105.7	28.5	103	5.3%	9.70 [1.92, 17.48]
Hanefeld 2011	120.26	26.3	39	119.1	30.16	42	3.8%	1.16 [-11.14, 13.46]
Kaku 2009	128.4	23.2	83	126.5	20	86	5.8%	1.90 [-4.64, 8.44]
Perez 2009	119.71	2.38	201	115.5	2.34	210	7.2%	4.21 [3.75, 4.67]
Wang 2006	138.9	52.1	24	140.5	37.1	12	1.2%	-1.60 [-31.18, 27.98]
**Subtotal (95% CI)**			**625**			**613**	**30.6%**	**2.22 [-3.15, 7.58]**
Heterogeneity: Tau² = 29.30; Chi² = 353.40, df = 5 (P < 0.00001); I² = 99%								
Test for overall effect: Z = 0.81 (P = 0.42)								
**Rosiglitazone**								
Bailey 2005	126	16.1	289	114.95	12.33	280	7.0%	11.05 [8.70, 13.40]
Borges 2011	106.99	15.14	348	99.2	15.96	340	7.0%	7.79 [5.46, 10.12]
Fonseca 2000 (high dose)	133.41	40.22	113	121.04	37.51	58	3.9%	12.37 [0.20, 24.54]
Fonseca 2000 (low dose)	133.8	33.26	119	121.04	37.51	58	4.1%	12.76 [1.41, 24.11]
Gomez-Perez 2002 (high dose)	123.5	25.7	40	115	27.7	20	3.2%	8.50 [-6.02, 23.02]
Gomez-Perez 2002 (low dose)	114.3	30	37	115	27.7	19	3.0%	-0.70 [-16.47, 15.07]
Kadoglou 2010	134.6	46.9	50	132.8	54.3	50	2.2%	1.80 [-18.09, 21.69]
Kadoglou 2011	135	50	70	126	47	70	2.9%	9.00 [-7.08, 25.08]
Rosenstock 2006	113.54	11.5	155	103.66	13.22	154	6.9%	9.88 [7.12, 12.64]
Scott 2008	119.6	37.6	87	108.4	33.6	92	4.4%	11.20 [0.73, 21.67]
Stewart 2006	129.346	12.81	254	115.23	13.4	272	7.0%	14.12 [11.88, 16.36]
Weissman 2005	118.56	13.15	382	101.64	11.57	384	7.1%	16.92 [15.17, 18.67]
**Subtotal (95% CI)**			**1944**			**1797**	**58.7%**	**11.30 [8.46, 14.14]**
Heterogeneity: Tau² = 12.44; Chi² = 51.30, df = 11 (P < 0.00001); I² = 79%								
Test for overall effect: Z = 7.80 (P < 0.00001)								
**Tesaglitazar**								
Goke 2007 (high dose)	103.64	34.8	196	108.28	30.94	100	5.3%	-4.64 [-12.42, 3.14]
Goke 2007 (low dose)	110.21	34.8	194	108.28	30.94	100	5.3%	1.93 [-5.86, 9.72]
**Subtotal (95% CI)**			**390**			**200**	**10.7%**	**-1.36 [-7.80, 5.08]**
Heterogeneity: Tau² = 5.80; Chi² = 1.37, df = 1 (P = 0.24); I² = 27%								
Test for overall effect: Z = 0.41 (P = 0.68)								
**Total (95% CI)**			**2959**			**2610**	**100.0%**	**6.81 [3.28, 10.33]**
Heterogeneity: Tau² = 44.81; Chi² = 826.35, df = 19 (P < 0.00001); I² = 98%								
Test for overall effect: Z = 3.78 (P = 0.0002)								
Test for subgroup differences: Chi² = 17.77, df = 2 (P = 0.0001), I² = 88.7%								

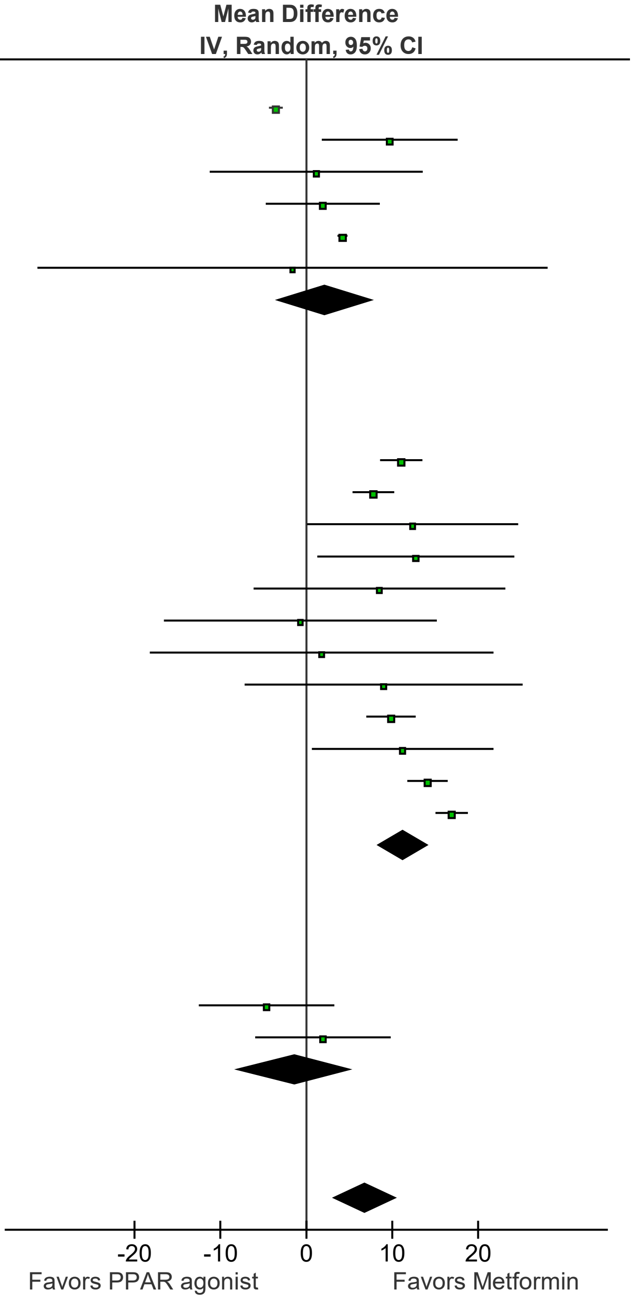

Supplement Figure 9. Triglycerides

	PPAR agonist plus metformin			Metformin alone				Mean Difference
**Study or Subgroup**	**Mean**	**SD**	**Total**	**Mean**	**SD**	**Total**	**Weight**	**IV, Random, 95% CI**
**Pioglitazone**								
Einhorn 2000	269.91	24.9	168	325.93	25.86	160	6.3%	-56.02 [-61.52, -50.52]
Genovese 2013	158.4	90.5	110	165.8	91.5	103	4.9%	-7.40 [-31.86, 17.06]
Hanefeld 2011	136.4	55.8	39	155.88	62	42	4.8%	-19.48 [-45.14, 6.18]
Kaku 2009	151.1	76.3	83	140.3	93.8	86	4.8%	10.80 [-14.94, 36.54]
Perez 2009	146.79	24.01	201	148.83	19.34	210	6.3%	-2.04 [-6.27, 2.19]
Wang 2006	75.2	51.7	24	135.4	86	12	2.6%	-60.20 [-113.07, -7.33]
**Subtotal (95% CI)**			**625**			**613**	**29.6%**	**-20.87 [-51.14, 9.39]**
Heterogeneity: Tau² = 1257.54; Chi² = 242.27, df = 5 (P < 0.00001); I² = 98%								
Test for overall effect: Z = 1.35 (P = 0.18)								
**Rosiglitazone**								
Bailey 2005	189.73	31.63	289	167.79	28.62	280	6.3%	21.94 [16.99, 26.89]
Borges 2011	80.85	21.93	348	81.3	20.64	340	6.3%	-0.45 [-3.63, 2.73]
Fonseca 2000 (high dose)	227.62	165.63	113	246.22	158.54	58	2.7%	-18.60 [-69.56, 32.36]
Fonseca 2000 (low dose)	232.05	139.05	119	246.22	158.54	58	2.9%	-14.17 [-62.01, 33.67]
Gomez-Perez 2002 (high dose)	199.9	113.3	40	233.4	126.8	20	2.0%	-33.50 [-99.23, 32.23]
Gomez-Perez 2002 (low dose)	193.8	133.2	37	233.4	126.8	19	1.8%	-39.60 [-110.96, 31.76]
Kadoglou 2010	164.2	114.6	50	115.2	40.4	50	4.0%	49.00 [15.32, 82.68]
Kadoglou 2011	154	55	70	114	36	70	5.7%	40.00 [24.60, 55.40]
Negro 2005	127	41.4	19	161	44.4	19	4.6%	-34.00 [-61.30, -6.70]
Rosenstock 2006	199.8	108.4	155	191.5	111.1	154	4.9%	8.30 [-16.18, 32.78]
Scott 2008	79.74	20.62	87	75.5	19.91	92	6.3%	4.24 [-1.70, 10.18]
Stewart 2006	190.26	10.17	254	209.9	9.95	272	6.4%	-19.64 [-21.36, -17.92]
Weissman 2005	197.05	36.72	382	177.12	31.06	384	6.3%	19.93 [15.11, 24.75]
**Subtotal (95% CI)**			**1963**			**1816**	**60.1%**	**4.42 [-8.84, 17.68]**
Heterogeneity: Tau² = 416.97; Chi² = 537.91, df = 12 (P < 0.00001); I² = 98%								
Test for overall effect: Z = 0.65 (P = 0.51)								
**Tesaglitazar**								
Goke 2007 (high dose)	68.2	81.48	196	162.17	88.57	100	5.2%	-93.97 [-114.74, -73.20]
Goke 2007 (low dose)	113.37	95.66	194	162.08	88.57	100	5.1%	-48.71 [-70.68, -26.74]
**Subtotal (95% CI)**			**390**			**200**	**10.3%**	**-71.49 [-115.84, -27.13]**
Heterogeneity: Tau² = 905.27; Chi² = 8.61, df = 1 (P = 0.003); I² = 88%								
Test for overall effect: Z = 3.16 (P = 0.002)								
**Total (95% CI)**			**2978**			**2629**	**100.0%**	**-10.96 [-22.10, 0.18]**
Heterogeneity: Tau² = 507.55; Chi² = 906.79, df = 20 (P < 0.00001); I² = 98%								
Test for overall effect: Z = 1.93 (P = 0.05)								
Test for subgroup differences: Chi² = 11.62, df = 2 (P = 0.003), I² = 82.8%								

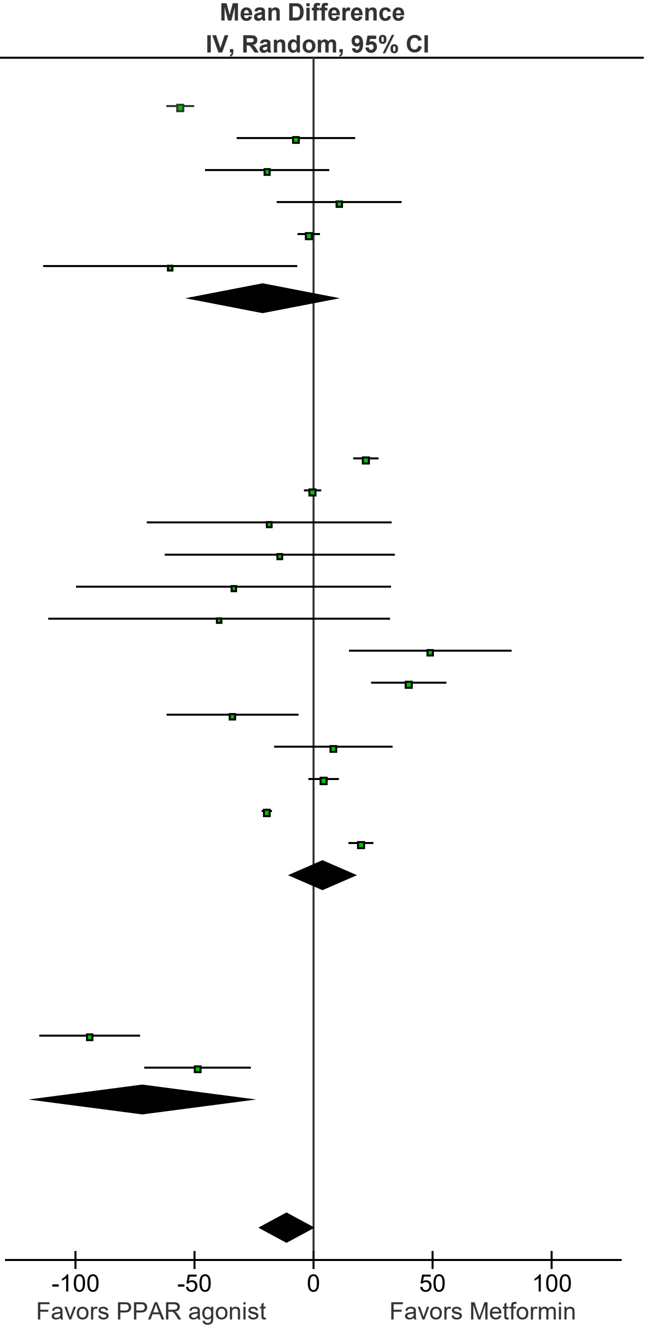

Supplement Figure 10. Systolic blood pressure

Study or Subgroup	PPAR agonist plus metformin Mean	SD	Total	Metformin alone Mean	SD	Total	Weight	Mean Difference IV, Random, 95% CI
**Pioglitazone**								
Genovese 2013	129.3	14.7	110	132.1	15.9	103	12.7%	-2.80 [-6.92, 1.32]
Wang 2006	110	9	24	116	7	12	8.2%	-6.00 [-11.35, -0.65]
**Subtotal (95% CI)**			**134**			**115**	**20.9%**	**-3.99 [-7.26, -0.73]**
Heterogeneity: Tau² = 0.00; Chi² = 0.86, df = 1 (P = 0.35); I² = 0%								
Test for overall effect: Z = 2.40 (P = 0.02)								
**Rosiglitazone**								
Kadoglou 2010	130.6	17.4	50	133	15.6	50	5.8%	-2.40 [-8.88, 4.08]
Kadoglou 2011	129	19	70	135	19	70	6.1%	-6.00 [-12.29, 0.29]
Negro 2005	131.6	2.8	19	133	2.7	19	37.4%	-1.40 [-3.15, 0.35]
Stewart 2006	127.6	12	254	132	13.9	272	29.7%	-4.40 [-6.62, -2.18]
**Subtotal (95% CI)**			**393**			**411**	**79.1%**	**-3.06 [-5.22, -0.90]**
Heterogeneity: Tau² = 1.98; Chi² = 5.45, df = 3 (P = 0.14); I² = 45%								
Test for overall effect: Z = 2.78 (P = 0.005)								
**Total (95% CI)**			**527**			**526**	**100.0%**	**-3.19 [-4.83, -1.55]**
Heterogeneity: Tau² = 1.07; Chi² = 6.83, df = 5 (P = 0.23); I² = 27%								
Test for overall effect: Z = 3.81 (P = 0.0001)								
Test for subgroup differences: Chi² = 0.22, df = 1 (P = 0.64), I² = 0%								

Mean Difference
IV, Random, 95% CI

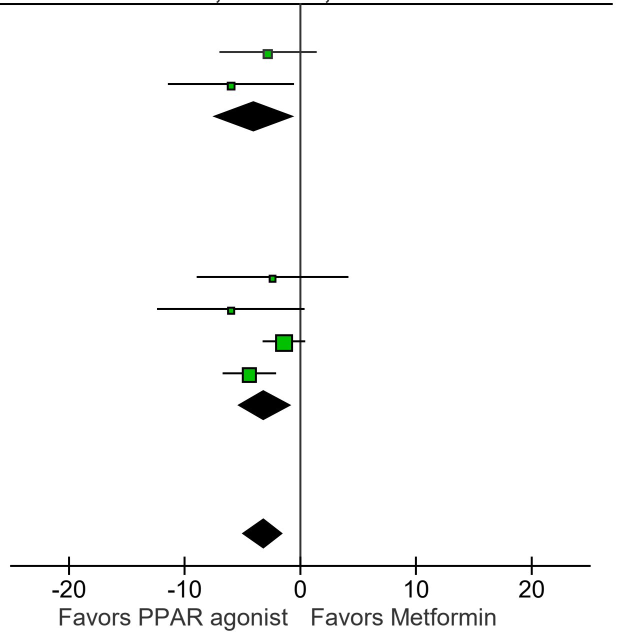

Supplement Figure 11. Diastolic blood pressure

Study or Subgroup	PPAR agonist plus metformin Mean	SD	Total	Metformin alone Mean	SD	Total	Weight	Mean Difference IV, Random, 95% CI
**Pioglitazone**								
Genovese 2013	79.8	7.7	110	80.7	8.3	103	18.3%	-0.90 [-3.05, 1.25]
Wang 2006	68	8	24	72	7	12	10.0%	-4.00 [-9.09, 1.09]
**Subtotal (95% CI)**			**134**			**115**	**28.3%**	**-1.56 [-4.04, 0.93]**
Heterogeneity: Tau² = 0.83; Chi² = 1.21, df = 1 (P = 0.27); I² = 17%								
Test for overall effect: Z = 1.23 (P = 0.22)								
**Rosiglitazone**								
Kadoglou 2010	77.5	9.5	50	79.3	5.6	50	15.5%	-1.80 [-4.86, 1.26]
Kadoglou 2011	79	9	70	81	8	70	16.2%	-2.00 [-4.82, 0.82]
Negro 2005	77.4	2.7	19	84	2.5	19	19.7%	-6.60 [-8.25, -4.95]
Stewart 2006	73.9	8.5	254	75.6	8.6	272	20.3%	-1.70 [-3.16, -0.24]
**Subtotal (95% CI)**			**393**			**411**	**71.7%**	**-3.12 [-5.92, -0.32]**
Heterogeneity: Tau² = 6.81; Chi² = 21.56, df = 3 (P < 0.0001); I² = 86%								
Test for overall effect: Z = 2.18 (P = 0.03)								
**Total (95% CI)**			**527**			**526**	**100.0%**	**-2.82 [-4.99, -0.64]**
Heterogeneity: Tau² = 5.51; Chi² = 26.09, df = 5 (P < 0.0001); I² = 81%								
Test for overall effect: Z = 2.54 (P = 0.01)								
Test for subgroup differences: Chi² = 0.67, df = 1 (P = 0.41), I² = 0%								

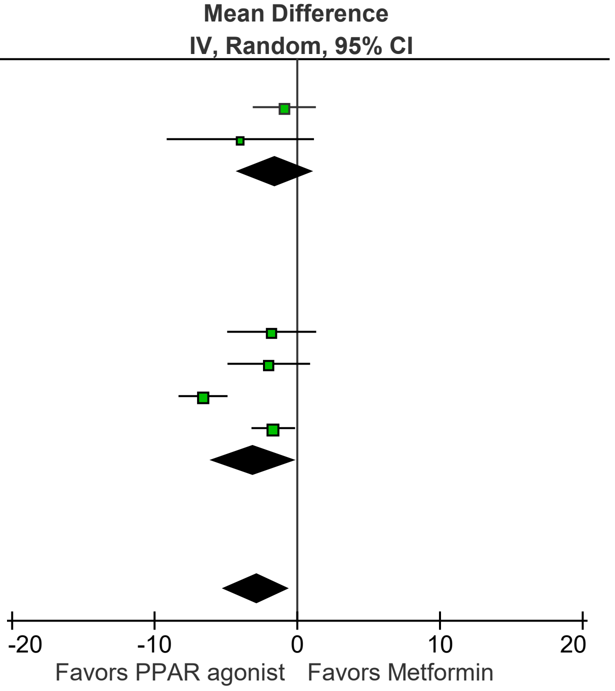

Supplement Figure 12. Gastrointestinal adverse events

	PPAR agonist plus metformin		Metformin alone			Risk Ratio
**Study or Subgroup**	**Events**	**Total**	**Events**	**Total**	**Weight**	**M-H, Random, 95% CI**
**Pioglitazone**						
Genovese 2013	15	97	23	97	3.1%	0.65 [0.36, 1.17]
Perez 2009	22	201	39	209	4.5%	0.59 [0.36, 0.95]
**Subtotal (95% CI)**		**298**		**306**	**7.6%**	**0.61 [0.42, 0.89]**
Total events	37		62			
Heterogeneity: Tau² = 0.00; Chi² = 0.07, df = 1 (P = 0.78); I² = 0%						
Test for overall effect: Z = 2.57 (P = 0.01)						
**Rosiglitazone**						
Bailey 2005	64	258	74	236	13.1%	0.79 [0.60, 1.05]
Borges 2011	72	344	93	334	14.7%	0.75 [0.57, 0.98]
Rosenstock 2006	73	155	79	154	20.6%	0.92 [0.73, 1.15]
Scott 2008	6	87	8	91	1.0%	0.78 [0.28, 2.17]
Stewart 2006	84	254	90	272	17.9%	1.00 [0.78, 1.27]
Weissman 2005	100	358	136	351	23.5%	0.72 [0.58, 0.89]
**Subtotal (95% CI)**		**1456**		**1438**	**90.9%**	**0.83 [0.74, 0.93]**
Total events	399		480			
Heterogeneity: Tau² = 0.00; Chi² = 5.37, df = 5 (P = 0.37); I² = 7%						
Test for overall effect: Z = 3.24 (P = 0.001)						
**Tesaglitazar**						
Goke 2007 (high dose)	7	194	4	99	0.7%	0.89 [0.27, 2.98]
Goke 2007 (low dose)	7	193	5	99	0.8%	0.72 [0.23, 2.20]
**Subtotal (95% CI)**		**387**		**198**	**1.6%**	**0.79 [0.35, 1.81]**
Total events	14		9			
Heterogeneity: Tau² = 0.00; Chi² = 0.07, df = 1 (P = 0.80); I² = 0%						
Test for overall effect: Z = 0.55 (P = 0.58)						
**Total (95% CI)**		**2141**		**1942**	**100.0%**	**0.81 [0.73, 0.90]**
Total events	450		551			
Heterogeneity: Tau² = 0.00; Chi² = 7.90, df = 9 (P = 0.54); I² = 0%						
Test for overall effect: Z = 4.01 (P < 0.0001)						
Test for subgroup differences: Chi² = 2.32, df = 2 (P = 0.31), I² = 13.9%						

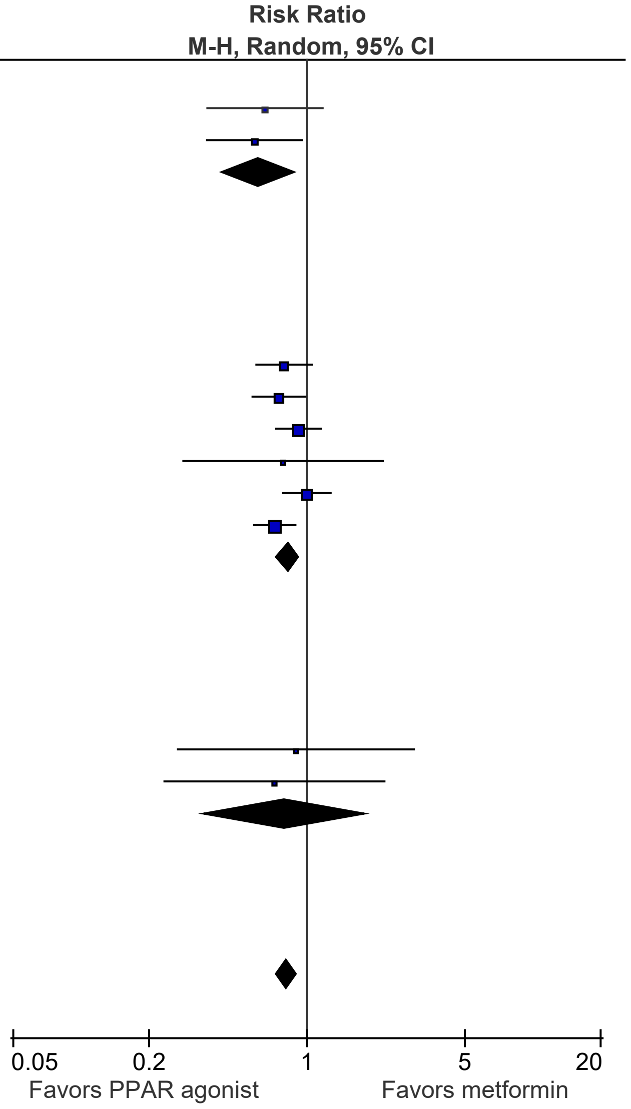

Supplement Figure 13. Subgroup analysis by PPAR agonist dose for the outcome of fasting blood glucose

Study or Subgroup	PPAR agonist plus metform Mean	SD	Total	Metformin alone Mean	SD	Total	Weight	Mean Difference IV, Random, 95% CI
**PPAR Agonist Dose <15 mg/day**								
Bailey 2005	144.1	33	289	164	37	280	5.7%	-19.90 [-25.67, -14.13]
Borges 2011	120.6	54	348	138.6	59.4	340	5.2%	-18.00 [-26.49, -9.51]
Fonseca 2000 (high dose)	171.02	54.9	113	219.546	52.38	58	3.7%	-48.53 [-65.38, -31.67]
Fonseca 2000 (low dose)	181.206	57.06	119	219.546	52.38	58	3.7%	-38.34 [-55.28, -21.40]
Goke 2007 (high dose)	129.6	31.14	196	161.1	31.32	100	5.4%	-31.50 [-39.03, -23.97]
Goke 2007 (low dose)	143.46	31.14	194	161.1	31.32	100	5.4%	-17.64 [-25.18, -10.10]
Gomez-Perez 2002 (high dose)	171	65	40	228	58.3	20	1.8%	-57.00 [-89.54, -24.46]
Gomez-Perez 2002 (low dose)	190	65	37	228	58.3	19	1.7%	-38.00 [-71.55, -4.45]
Kadoglou 2010	145.5	43.2	50	152.6	25.7	50	4.2%	-7.10 [-21.03, 6.83]
Kadoglou 2011	142	38	70	141	24	70	4.8%	1.00 [-9.53, 11.53]
Negro 2005	165.1	12.5	19	180	14.3	19	5.2%	-14.90 [-23.44, -6.36]
Rosenstock 2006	127.8	52.2	155	147.6	52.2	154	4.6%	-19.80 [-31.44, -8.16]
Scott 2008	132.8	29.9	87	165.4	50.2	92	4.6%	-32.60 [-44.63, -20.57]
Stewart 2006	128.88	31.68	254	138.24	33.12	272	5.7%	-9.36 [-14.90, -3.82]
Weissman 2005	134.1	52.38	382	150.48	48.6	384	5.5%	-16.38 [-23.54, -9.22]
**Subtotal (95% CI)**			**2353**			**2016**	**67.0%**	**-21.16 [-26.83, -15.48]**
Heterogeneity: Tau² = 87.08; Chi² = 68.40, df = 14 (P < 0.00001); I² = 80%								
Test for overall effect: Z = 7.31 (P < 0.00001)								
**PPAR Agonist Dose ≥ 15 mg/day**								
Derosa 2009	139	10	69	148	19	67	5.8%	-9.00 [-14.12, -3.88]
Einhorn 2000	209.3	69.7	168	253.6	68.6	160	4.0%	-44.30 [-59.27, -29.33]
Genovese 2013	130.5	28.5	110	149.5	35.5	103	5.2%	-19.00 [-27.68, -10.32]
Hanefeld 2011	117.36	26.64	39	131.76	34.2	42	4.3%	-14.40 [-27.70, -1.10]
Kaku 2009	138.1	28.7	83	161	30.1	86	5.2%	-22.90 [-31.77, -14.03]
Takeda 2008	133.032	47.6706	157	181.385	47.8604	158	4.8%	-48.35 [-58.90, -37.80]
Wang 2006	110	16	24	117	27	12	3.7%	-7.00 [-23.56, 9.56]
**Subtotal (95% CI)**			**650**			**628**	**33.0%**	**-23.44 [-35.06, -11.82]**
Heterogeneity: Tau² = 211.88; Chi² = 58.53, df = 6 (P < 0.00001); I² = 90%								
Test for overall effect: Z = 3.95 (P < 0.0001)								
**Total (95% CI)**			**3003**			**2644**	**100.0%**	**-22.07 [-27.17, -16.97]**

Heterogeneity: Tau² = 110.97; Chi² = 126.98, df = 21 (P < 0.00001); I² = 83%
Test for overall effect: Z = 8.48 (P < 0.00001)
Test for subgroup differences: Chi² = 0.12, df = 1 (P = 0.73), I² = 0%

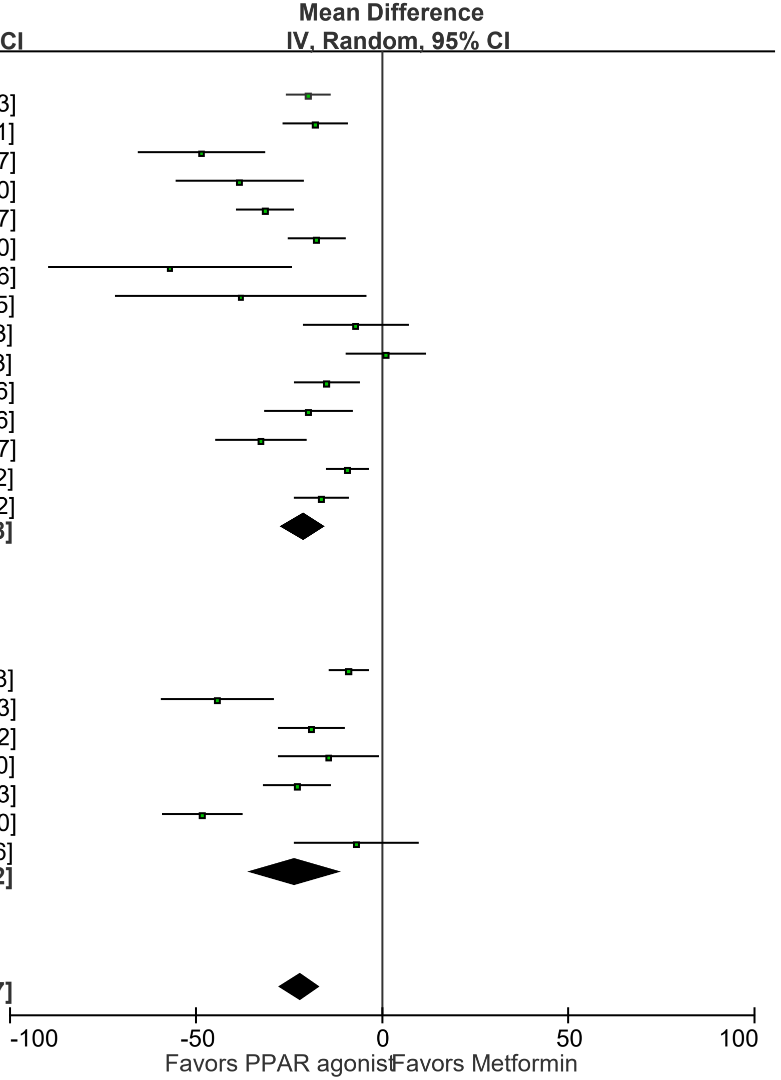

Supplement Figure 14. Subgroup analysis by PPAR agonist dose for the outcome of HBA1C concentration

	PPAR agonist plus metform			Metformin alone				Mean Difference
**Study or Subgroup**	**Mean**	**SD**	**Total**	**Mean**	**SD**	**Total**	**Weight**	**IV, Random, 95% CI**
**PPAR Agonist Dose <15 mg/day**								
Bailey 2005	7.1	1.1	289	7.4	1.1	280	5.6%	-0.30 [-0.48, -0.12]
Borges 2011	6.65	0.9	348	6.9	0.9	340	5.8%	-0.25 [-0.38, -0.12]
Fonseca 2000 (high dose)	8.12	1.5	113	9.05	1.3	58	3.9%	-0.93 [-1.36, -0.50]
Fonseca 2000 (low dose)	8.34	1.3	119	9.05	1.3	58	4.1%	-0.71 [-1.12, -0.30]
Goke 2007 (high dose)	6.89	0.67	196	7.66	0.69	100	5.7%	-0.77 [-0.93, -0.61]
Goke 2007 (low dose)	7.1	0.65	194	7.66	0.69	100	5.7%	-0.56 [-0.72, -0.40]
Gomez-Perez 2002 (high dose)	8.6	1.8	40	10.1	1.7	20	1.7%	-1.50 [-2.43, -0.57]
Gomez-Perez 2002 (low dose)	9.4	2.4	37	10.1	1.7	19	1.4%	-0.70 [-1.79, 0.39]
Kadoglou 2010	6.98	1.32	50	7.07	1.04	50	3.7%	-0.09 [-0.56, 0.38]
Kadoglou 2011	6.6	1.1	70	6.87	1.13	70	4.4%	-0.27 [-0.64, 0.10]
Negro 2005	7.3	0.7	19	8.3	0.5	19	4.2%	-1.00 [-1.39, -0.61]
Rosenstock 2006	6.6	1	155	7	1	154	5.4%	-0.40 [-0.62, -0.18]
Scott 2008	6.94	0.75	87	7.47	1.05	92	5.1%	-0.53 [-0.80, -0.26]
Stewart 2006	6.7	0.8	254	6.8	0.9	272	5.8%	-0.10 [-0.25, 0.05]
Weissman 2005	7.07	1.17	382	7.19	1.19	384	5.7%	-0.12 [-0.29, 0.05]
**Subtotal (95% CI)**			**2353**			**2016**	**68.1%**	**-0.47 [-0.62, -0.32]**
Heterogeneity: Tau² = 0.06; Chi² = 81.66, df = 14 (P < 0.00001); I² = 83%								
Test for overall effect: Z = 5.98 (P < 0.00001)								
**PPAR Agonist Dose ≥ 15 mg/day**								
Derosa 2009	7.2	0.3	69	7.9	0.5	67	5.8%	-0.70 [-0.84, -0.56]
Einhorn 2000	9.8	1.4	168	9.95	1.3	160	4.9%	-0.15 [-0.44, 0.14]
Genovese 2013	6.41	0.65	110	6.96	0.74	103	5.6%	-0.55 [-0.74, -0.36]
Hanefeld 2011	6.85	0.75	39	7.23	0.66	42	4.8%	-0.38 [-0.69, -0.07]
Kaku 2009	6.91	0.8	83	7.8	0.92	86	5.1%	-0.89 [-1.15, -0.63]
Takeda 2008	7.48	0.7244	157	8.51	0.7244	158	5.7%	-1.03 [-1.19, -0.87]
**Subtotal (95% CI)**			**626**			**616**	**31.9%**	**-0.63 [-0.87, -0.40]**
Heterogeneity: Tau² = 0.07; Chi² = 38.53, df = 5 (P < 0.00001); I² = 87%								
Test for overall effect: Z = 5.30 (P < 0.00001)								
**Total (95% CI)**			**2979**			**2632**	**100.0%**	**-0.53 [-0.67, -0.38]**
Heterogeneity: Tau² = 0.09; Chi² = 163.82, df = 20 (P < 0.00001); I² = 88%								
Test for overall effect: Z = 7.21 (P < 0.00001)								
Test for subgroup differences: Chi² = 1.30, df = 1 (P = 0.25), I² = 23.3%								

Mean Difference
IV, Random, 95% CI

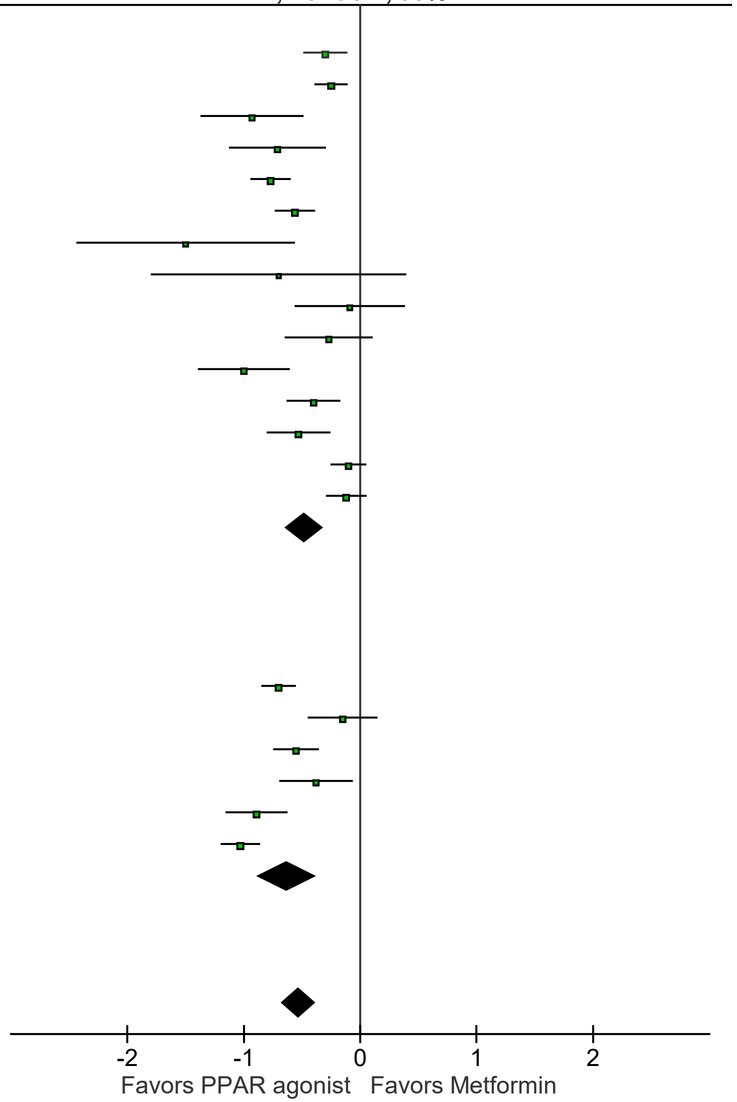

Supplement Figure 15. Subgroup analysis by a priori power analysis performed or not for the outcome of fasting blood glucose

	PPAR agonist plus metform			Metformin alone				Mean Difference
**Study or Subgroup**	**Mean**	**SD**	**Total**	**Mean**	**SD**	**Total**	**Weight**	**IV, Random, 95% CI**
**A priori sample size calculations reported**								
Bailey 2005	144.1	33	289	164	37	280	5.7%	-19.90 [-25.67, -14.13]
Borges 2011	120.6	54	348	138.6	59.4	340	5.2%	-18.00 [-26.49, -9.51]
Derosa 2009	139	10	69	148	19	67	5.8%	-9.00 [-14.12, -3.88]
Fonseca 2000 (high dose)	171.02	54.9	113	219.546	52.38	58	3.7%	-48.53 [-65.38, -31.67]
Fonseca 2000 (low dose)	181.206	57.06	119	219.546	52.38	58	3.7%	-38.34 [-55.28, -21.40]
Goke 2007 (high dose)	129.6	31.14	196	161.1	31.32	100	5.4%	-31.50 [-39.03, -23.97]
Goke 2007 (low dose)	143.46	31.14	194	161.1	31.32	100	5.4%	-17.64 [-25.18, -10.10]
Gomez-Perez 2002 (high dose)	171	65	40	228	58.3	20	1.8%	-57.00 [-89.54, -24.46]
Gomez-Perez 2002 (low dose)	190	65	37	228	58.3	19	1.7%	-38.00 [-71.55, -4.45]
Hanefeld 2011	117.36	26.64	39	131.76	34.2	42	4.3%	-14.40 [-27.70, -1.10]
Kaku 2009	138.1	28.7	83	161	30.1	86	5.2%	-22.90 [-31.77, -14.03]
Rosenstock 2006	127.8	52.2	155	147.6	52.2	154	4.6%	-19.80 [-31.44, -8.16]
Stewart 2006	128.88	31.68	254	138.24	33.12	272	5.7%	-9.36 [-14.90, -3.82]
Weissman 2005	134.1	52.38	382	150.48	48.6	384	5.5%	-16.38 [-23.54, -9.22]
**Subtotal (95% CI)**			**2318**			**1980**	**63.4%**	**-21.65 [-26.97, -16.33]**
Heterogeneity: Tau² = 68.86; Chi² = 60.62, df = 13 (P < 0.00001); I² = 79%								
Test for overall effect: Z = 7.98 (P < 0.00001)								
**No A priori sample size calculations reported**								
Einhorn 2000	209.3	69.7	168	253.6	68.6	160	4.0%	-44.30 [-59.27, -29.33]
Genovese 2013	130.5	28.5	110	149.5	35.5	103	5.2%	-19.00 [-27.68, -10.32]
Kadoglou 2010	145.5	43.2	50	152.6	25.7	50	4.2%	-7.10 [-21.03, 6.83]
Kadoglou 2011	142	38	70	141	24	70	4.8%	1.00 [-9.53, 11.53]
Negro 2005	165.1	12.5	19	180	14.3	19	5.2%	-14.90 [-23.44, -6.36]
Scott 2008	132.8	29.9	87	165.4	50.2	92	4.6%	-32.60 [-44.63, -20.57]
Takeda 2008	133.032	47.6706	157	181.385	47.8604	158	4.8%	-48.35 [-58.90, -37.80]
Wang 2006	110	16	24	117	27	12	3.7%	-7.00 [-23.56, 9.56]
**Subtotal (95% CI)**			**685**			**664**	**36.6%**	**-21.55 [-33.69, -9.40]**
Heterogeneity: Tau² = 268.67; Chi² = 64.21, df = 7 (P < 0.00001); I² = 89%								
Test for overall effect: Z = 3.48 (P = 0.0005)								
**Total (95% CI)**			**3003**			**2644**	**100.0%**	**-22.07 [-27.17, -16.97]**
Heterogeneity: Tau² = 110.97; Chi² = 126.98, df = 21 (P < 0.00001); I² = 83%								
Test for overall effect: Z = 8.48 (P < 0.00001)								
Test for subgroup differences: Chi² = 0.00, df = 1 (P = 0.99), I² = 0%								

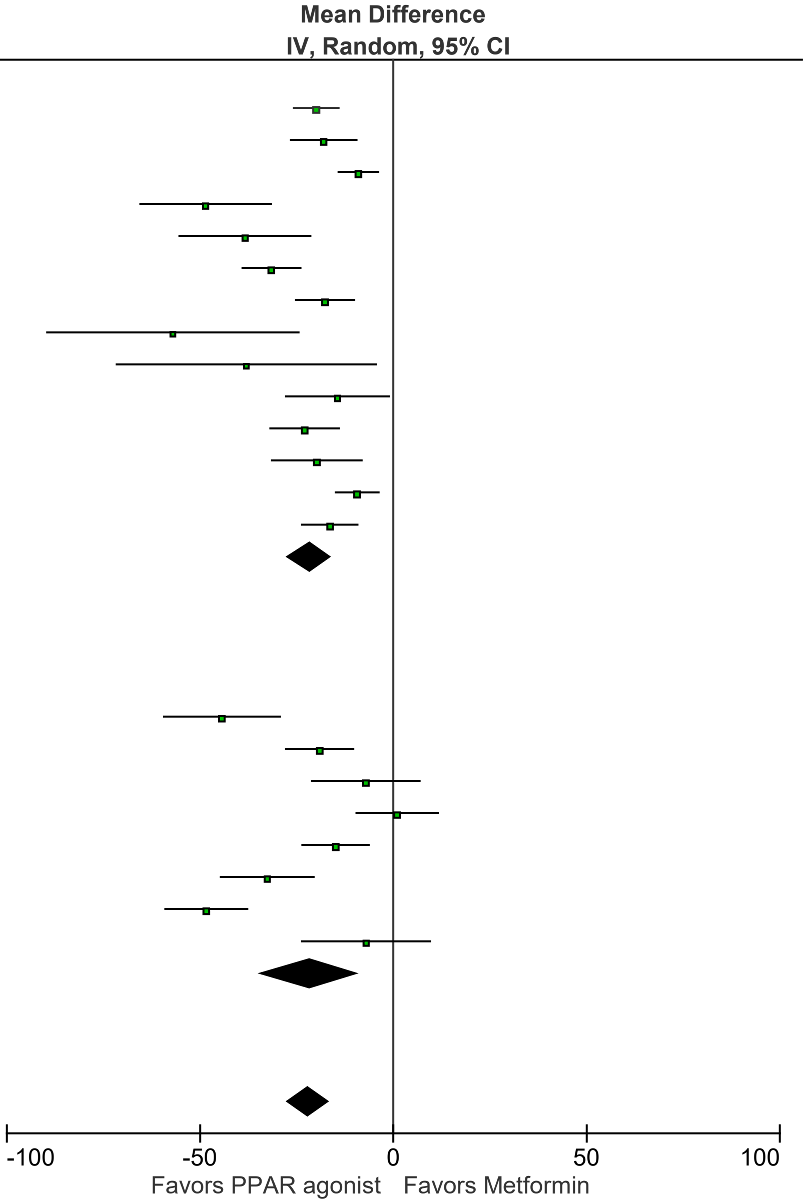

Supplement Figure 16. Subgroup analysis by a priori power analysis performed or not for the outcome of HBA1C concentration

	PPAR agonist plus metform			Metformin alone				Mean Difference
**Study or Subgroup**	**Mean**	**SD**	**Total**	**Mean**	**SD**	**Total**	**Weight**	**IV, Random, 95% CI**
**A priori sample size calculations reported**								
Bailey 2005	7.1	1.1	289	7.4	1.1	280	5.6%	-0.30 [-0.48, -0.12]
Borges 2011	6.65	0.9	348	6.9	0.9	340	5.8%	-0.25 [-0.38, -0.12]
Derosa 2009	7.2	0.3	69	7.9	0.5	67	5.8%	-0.70 [-0.84, -0.56]
Fonseca 2000 (high dose)	8.12	1.5	113	9.05	1.3	58	3.9%	-0.93 [-1.36, -0.50]
Fonseca 2000 (low dose)	8.34	1.3	119	9.05	1.3	58	4.1%	-0.71 [-1.12, -0.30]
Goke 2007 (high dose)	6.89	0.67	196	7.66	0.69	100	5.7%	-0.77 [-0.93, -0.61]
Goke 2007 (low dose)	7.1	0.65	194	7.66	0.69	100	5.7%	-0.56 [-0.72, -0.40]
Gomez-Perez 2002 (high dose)	8.6	1.8	40	10.1	1.7	20	1.7%	-1.50 [-2.43, -0.57]
Gomez-Perez 2002 (low dose)	9.4	2.4	37	10.1	1.7	19	1.4%	-0.70 [-1.79, 0.39]
Hanefeld 2011	6.85	0.75	39	7.23	0.66	42	4.8%	-0.38 [-0.69, -0.07]
Kaku 2009	6.91	0.8	83	7.8	0.92	86	5.1%	-0.89 [-1.15, -0.63]
Rosenstock 2006	6.6	1	155	7	1	154	5.4%	-0.40 [-0.62, -0.18]
Stewart 2006	6.7	0.8	254	6.8	0.9	272	5.8%	-0.10 [-0.25, 0.05]
Weissman 2005	7.07	1.17	382	7.19	1.19	384	5.7%	-0.12 [-0.29, 0.05]
**Subtotal (95% CI)**			**2318**			**1980**	**66.4%**	**-0.52 [-0.68, -0.35]**
Heterogeneity: Tau² = 0.07; Chi² = 99.59, df = 13 (P < 0.00001); I² = 87%								
Test for overall effect: Z = 6.23 (P < 0.00001)								
**No A priori sample size calculations reported**								
Einhorn 2000	9.8	1.4	168	9.95	1.3	160	4.9%	-0.15 [-0.44, 0.14]
Genovese 2013	6.41	0.65	110	6.96	0.74	103	5.6%	-0.55 [-0.74, -0.36]
Kadoglou 2010	6.98	1.32	50	7.07	1.04	50	3.7%	-0.09 [-0.56, 0.38]
Kadoglou 2011	6.6	1.1	70	6.87	1.13	70	4.4%	-0.27 [-0.64, 0.10]
Negro 2005	7.3	0.7	19	8.3	0.5	19	4.2%	-1.00 [-1.39, -0.61]
Scott 2008	6.94	0.75	87	7.47	1.05	92	5.1%	-0.53 [-0.80, -0.26]
Takeda 2008	7.48	0.7244	157	8.51	0.7244	158	5.7%	-1.03 [-1.19, -0.87]
**Subtotal (95% CI)**			**661**			**652**	**33.6%**	**-0.53 [-0.82, -0.25]**
Heterogeneity: Tau² = 0.12; Chi² = 47.48, df = 6 (P < 0.00001); I² = 87%								
Test for overall effect: Z = 3.66 (P = 0.0003)								
**Total (95% CI)**			**2979**			**2632**	**100.0%**	**-0.53 [-0.67, -0.38]**
Heterogeneity: Tau² = 0.09; Chi² = 163.82, df = 20 (P < 0.00001); I² = 88%								
Test for overall effect: Z = 7.21 (P < 0.00001)								
Test for subgroup differences: Chi² = 0.01, df = 1 (P = 0.91), I² = 0%								

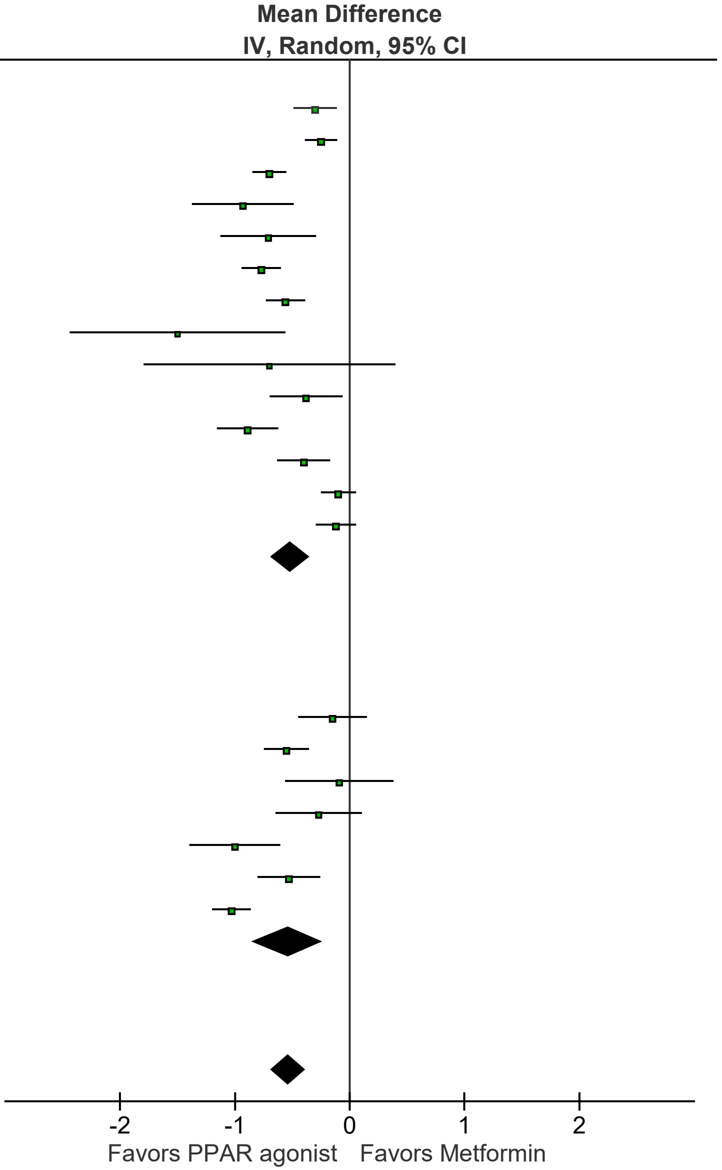

Supplement: Supplementary file 1 — Supplementary Information. [file 41598_2024_59390_MOESM1_ESM.pdf]
